# Application of the RE-AIM framework to evaluate the implementation of telehealth pulmonary rehabilitation in a randomized controlled trial among African-American and Hispanic patients with advanced stage Chronic Obstructive Pulmonary Disease

**DOI:** 10.1186/s12913-023-09492-7

**Published:** 2023-05-23

**Authors:** Jennifer Polo, Melissa J. Basile, Meng Zhang, Keyla Ordonez, Danielle Rodriguez, Eugenia Boye-Codjoe, Myia Williams, Donna Tsang, Richard Medina, Sonia Jacome, Parvez Mir, Sameer Khanijo, Renee Pekmezaris, Negin Hajizadeh

**Affiliations:** 1 Northwell Health, Great Neck, NY USA; 2 Institute of Health System Science, Northwell Health, 600 Community Drive, Suite 403, Manhasset, NY 11030 USA; 3 Department of Medicine, Donald and Barbara Zucker School of Medicine at Hofstra/Northwell, Manhasset, NY USA; 4 Wyckoff Heights Medical Center, Brooklyn, NY USA; 5 Department of Occupational Medicine, Epidemiology, and Prevention, Donald and Barbara Zucker School of Medicine at Hofstra/Northwell, Great Neck, NY USA

**Keywords:** Chronic obstructive pulmonary disease, Implementation, Pulmonary rehabilitation, Quality of life, Telehealth

## Abstract

**Background:**

Pulmonary rehabilitation (PR) decreases rehospitalization for people with COPD. However, less than 2% receive PR, partly due to lack of referral and sparsity of PR facilities. This disparity is particularly pronounced in African American and Hispanic persons with COPD. Telehealth-provided PR could increase access and improve health outcomes.

**Methods:**

We applied the RE-AIM framework in a *post-hoc* analysis of our mixed methods RCT comparing referral to Telehealth-delivered PR (TelePR) versus standard PR (SPR) for African American and Hispanic COPD patients hospitalized for COPD exacerbation. Both arms received a referral to PR for 8 weeks, social worker follow-up, and surveys administered at baseline, 8 weeks, 6, and 12 months. PR sessions were conducted twice a week for 90 min each (16 sessions total). Quantitative data were analyzed using 2-sample *t* tests or nonparametric Wilcoxon tests for continuous data and χ^2^/Fisher exact tests for categorical data. Logistic regression–estimated odds ratios (ORs) were used for the intention-to-treat primary outcome. Qualitative interviews were conducted at the end of the study to assess adherence and satisfaction and were analyzed using inductive and deductive methods. The goal was to understand Reach (whether the target population was able to be enrolled), Effectiveness (primary outcome was a composite of 6-month COPD rehospitalization and death), Adoption (proportion of people willing to initiate the program), Implementation (whether the program was able to be executed as intended, and Maintenance (whether the program was continued).

**Results:**

Two hundred nine people enrolled out of a 276-recruitment goal. Only 85 completed at least one PR session 57/111 (51%) TelePR; 28/98 (28%) SPR. Referral to TelePR compared to SPR did not decrease the composite outcome of 6-month COPD-readmission rate/death (OR1.35;95%CI 0.69,2.66). There was significant reduction in fatigue (PROMIS® scale) from baseline to 8-weeks in TelePR compared to SPR (MD-1.34; ± SD4.22; *p* = 0.02). Participants who received TelePR experienced improvements from baseline in several outcomes (ie, before and after 8 weeks of PR) in the following: COPD symptoms, knowledge about COPD management, fatigue, and functional capacity. Among the patients who had 1 initial visit, adherence rates were similar (TelePR arm, 59% of sessions; SPR arm, 63%). No intervention-related adverse events occurred. Barriers to PR adoption included difficulty or reluctance to complete medical clearances and beliefs about PR efficacy. Notably, only 9 participants sustained exercise after program completion. Maintenance of the program was not possible due to low insurance reimbursement and sparsity of Respiratory Therapists.

**Conclusions:**

TelePR can reach COPD patients with health disparities and can be successfully implemented. The small sample size and large confidence intervals prevent conclusion about the relative effectiveness of participating in TelePR compared to SPR. However, improved outcomes were seen for those in TelePR as well as in SPR. Increasing adoption of PR and TelePR requires consideration of comorbidity burden, and perception of PR utility, and must facilitate medical clearances. Given the sparsity of SPR locations, TelePR can overcome at least the barrier of access. However, given the challenges to the uptake and completion of PR - many of the additional barriers in PR (both in TelePR and SPR) need to be addressed. Awareness of these real-world challenges will not only inform implementation of TelePR for clinicians seeking to adopt this platform but will also inform study designers and reviewers regarding the feasibility of approaches to patient recruitment and retention.

**Supplementary Information:**

The online version contains supplementary material available at 10.1186/s12913-023-09492-7.

## Contribution to the literature

- Given the sparsity of physical PR locations, telehealth-delivered PR can overcome some barriers to access such as transportation and patient level mobility
- Although facilitating access to PR allowed us to control for common socioeconomic factors that typically prevent populations experiencing health/health care disparities from completing PR, decision-making about adherence was motivated by patients’ weighing their immediate short-term circumstances, comorbid disease demands, and nonbiomedical knowledge.
- To promote uptake, pulmonary rehabilitation referrals must include considerations of comorbidity burden, the perception of PR utility, and must facilitate medical clearances.

## Introduction

Chronic Obstructive Pulmonary Disease (COPD) is the leading cause of hospitalization for older adults in the United States, and accounts for 1.5 million emergency department visits per year [1]. Pulmonary Rehabilitation (PR) is recognized as a core component of the management of individuals with chronic respiratory disease [2], and has been shown to improve 1-year survival when initiated within 90-days after hospital discharge for COPD exacerbation [3]. However, less than 1.5% of all individuals with COPD participate in PR [4, 5]. Barriers include unawareness about PR benefits, lack of referral from primary providers, financial burden, limited PR sites, and barriers related to transportation to PR locations.

We hypothesized that referral to a telehealth-delivered PR program (TelePR) would overcome access barriers to PR, which in turn would increase adherence, with subsequent improvement in health outcomes. As with access to other forms of health care, people with health disparities also have barriers to accessing PR which include socioeconomic factors such as insurance coverage and transportation costs, disruption to established routines, travel and location of PR, lack of perceived benefit, depression and poor state of health, and inconvenient PR schedule times [6]. Therefore, a TelePR program would be particularly effective for African American and Hispanic people with COPD living in the New York City metropolitan region. We tested this hypothesis by adapting a standard PR program (SPR) to be delivered via telehealth (TelePR). Full methods described herein.

Despite iterative and user-centered design, and despite the iterative feedback of a stakeholder-based community advisory board, the study was limited by difficulties in recruitment and retention of the target population. To evaluate our TelePR program to inform future study designs and the implementation of TelePR, we applied The RE-AIM (reach, effectiveness, adoption, implementation, maintenance) framework. This systematic framework has been successfully used to evaluate the implementation of healthcare interventions [7, 8]. We also highlight areas that cannot be overcome by study design and represent structural and socio-economic barriers that are part of real-world studies.

## Methods

### Trial overview

This was a two-arm, mixed method, single-blinded superiority randomized controlled comparative effectiveness trial where we collected 12-month longitudinal data. The hypothesis was that a referral to TelePR would lead to lower 6-month rehospitalization, or death compared to a referral to standard, office-based pulmonary rehabilitation (SPR). The secondary outcomes included changes from baseline to post-PR sessions (i.e., day 1 and 8-weeks) and tertiary outcomes included update and adherence to PR after referral, longitudinal changes (6- and 12-months) in symptoms and whether patients would continue to exercise. Figure S1 provide overview of participant flow.

Outcomes were chosen based on the known effect of PR in COPD persons, and clinical relevance to patients and providers. Where available, we selected measures validated for use in both English/Spanish.

### Participants

Participants included African American or Hispanic persons who were hospitalized for a COPD exacerbation at one of 9 hospitals in the New York City metropolitan area. Eligibility criteria included: COPD diagnosis, Hispanic or African American ethnicity/race, and current hospitalization for COPD-exacerbation. Potential participants were identified via the electronic medical record.

### Consent process

Participants were invited to enroll in a program to assist with management of their COPD, a referral to SPR and survey follow-up over the course of 1 year. Participants were shown an English/Spanish testimonial video depicting SPR or TelePR during recruitment. If they agreed, they signed consent form 1 which allowed for prospective collection of data over the course of 1 year from the EMR and via phone call, as well as a referral to PR. Participants were then randomized to receive the PR component of the program via either (1) a referral to SPR or (2) a referral to TelePR. As per the modified Zelen Randomized Consent Form (MZRCF) method [9], only those randomized to TelePR were informed that TelePR was being offered instead of SPR and then invited to sign consent form 2 (Fig. S2). If the patient declined to sign consent form 2, they would be followed over the course of 1 year and analyzed in the TelePR arm, and the patient would receive a referral to SPR (per consent form 1).

The MZRCF method allows researchers to obtain consent from patient participants for longitudinal follow-up in standard of care and then to randomly assign study participants to either the intervention (TelePR) or the control arm (SPR), for which additional informed consent must be obtained for those enrolled in the technology-based arm (i.e., nonstandard arm, consent form 2). We predicted that if a study participant was randomly assigned in a conventional way and did not receive the “high-tech” TelePR intervention, it was likely that they would refuse to continue in the study.

After enrollment, participants needed to receive medical clearance to be able to participate in PR. This required confirmation of COPD by a pulmonary function test (PFT) or a pulmonologist’s clinical diagnosis and a medical provider’s determination that the patient could exercise safety. These determinations required an in-person visit to the patient’s pulmonologist and, for those with cardiac medical comorbidities, an additional visit to a cardiologist for medical clearance.

### Randomization

Randomization was carried out in permuted blocks and stratified by enrollment site and by race/ethnicity. Both the biostatistician performing outcomes analyses, and the clinicians who were providing medical clearance were blinded to study allocation/randomization.

### Intervention- TelePR

Participants in the TelePR arm had PR delivered via telehealth in either the participant’s home or community center (if preferred and depending on space in their homes). All the equipment including a full-size recumbent bicycle, weights, stretch bands, vital sign monitor and tablet computer with Wi-Fi card was delivered to patient homes. Prior to the first session, the RT met participants in their home/community center for training on device usage and to check oxygen supplementation devices. TelePR sessions were conducted by the RT with up to 3-participants simultaneously, via a secure HIPAA-compliant server using Zoom web-conferencing technology (Fig. S3). Participants received a Nonin® watch that transmitted vital signs directly to the platform for continuous monitoring during the PR sessions. A pulmonologist was on-call during each session in case of an emergency. The same educational videos used in SPR were shown while participants exercised. The exercise program (tracking, duration, progression etc.) was developed to parallel SPR.

After completion of 8-weeks of PR, all equipment was recovered from the TelePR participants. Both the TelePR and SPR participants were provided with an exercise-peddler and a list of community centers, gyms, and different SPR locations to encourage continuation of exercise.

### Active control- standard pulmonary rehabilitation

For those enrolled in the SPR arm, two SPR sites were made available to participants within the study geographic area: Northwell Health Physician Partners Pulmonary and Sleep Medicine at Lake Success, New York and Glen Cove Hospital Outpatient Pulmonary Rehabilitation Program, Glen Cove, New York. We recruited patients within the metropolitan New York area – depending on where the patients lived – these centers were either within a 20 min or 1 h commute for patients. SPR facilities are equipped with exercise equipment, vital sign monitors, and supplemental oxygen devices, and are staffed by a team of RTs, other clinicians, and administrators. Educational lectures are given as part of the PR sessions.

Participants in both arms received $175 for their time completing longitudinal surveys. TelePR was provided at no cost to the participant. However, participants in SPR were required to pay co-payments based on insurance laws and SPR was charged to participants’ health insurance.

TelePR was provided at no cost to the patient; however, SPR was charged to participants’ health insurance, and co-payments were required. Both arms needed to have medical clearance appointments submitted to their insurance carriers and to pay co-payments when applicable. During the initial consent (consent form 1), all participants were made aware of this real-world requirement for SPR. It was not until the participant was randomly assigned to TelePR that the research team explained that the intervention could not be paid for by insurance and therefore would be paid by the study grant. Before the start of the program, the social worker and research team had discussed sliding-scale payment options with the health system and worked closely with the medical billing departments to assist with insurance navigation for participants.

Transportation costs were covered to and from clinic visits for medical clearance appointments and the SPR sessions. When possible, the social worker attempted to leverage existing insurance-subsidized transportation programs to offset the cost to the patient.

### Exercise training content and progression for SPR and TelePR

Each PR session was approximately 60-min, consisting of 30-min of aerobic exercise on a treadmill (SPR) or bicycle (TelePR), 20-min of anaerobic exercise and 10-min of cool down. The bilingual respiratory therapist (RT) developed an individualized exercise program for each participant based on exercise capacity, and documented progress using standardized forms paralleling those used in SPR.

A bilingual social-worker maintained contact with each participant from the time of hospital discharge to completion of the PR program to assist with identifying insurance-subsidized transportation programs, insurance navigation for participants without insurance, and arranging clinic appointments.

### Outcome measures

The RCT outcome measures are presented within the RE-AIM (*Reach*,* Effectiveness*,* Adoption*,* Implementation* and *Maintenance*) framework below.

Table 1 provides a summary of the different dimensions of REAIM and the aspects that are being measured in the study.

**Table 1:** RE-AIM applied in context of TelePR vs SPR study

**Domain**	**Constructs**	**Outcomes Measured**
Reach	Ability to “reach” target population	Enrollment of target population
Effectiveness	Effect of intervention on outcomes of interest	Rehospitalization, mortality
Adoption	Proportion of people approached/willing to initiate intervention	Proportion of people willing to initiate telePR
Implementation	Ability to execute intervention as intended (fidelity)	Ability to deliver telePR at same standard as SPR
Maintenance	Continued use of intervention over time	Continuation of telePR and participant-level continuation of PR or other exercise

#### Reach

The Reach of an intervention measures whether the intended/target population was reached by the program, and whether the intended population expressed interest in participating in the program. This is outlined in the CONSORT diagram. To increase the likelihood that our intervention would appeal to our target population, we convened a study-specific Community Advisory Board (CAB) comprised of patients and caregiver’s representative of our target communities, directors of PR clinics, and clinicians. The CAB provided input on initial study protocols, modifications to increase recruitment and retention, and the acceptability of the equipment that was used in TelePR including testing the bikes and technologies associated. Additionally, as part of the clinical trial, we conducted interviews and focus groups with participants who had different levels of adherence to PR to identify and address barriers they encountered.

#### Effectiveness

##### Primary outcome

Our primary outcome was a composite of COPD-related hospital readmissions/death within 6-months of discharge, based on the mean duration of 6-month follow-up in the systematic review used for sample-size calculation [10].

##### Secondary outcomes

Our secondary outcomes included changes from baseline to post-PR sessions (i.e., day 1 and 8-weeks) in: perceived symptom control and Quality of Life (QOL) (CAT, MMRC) [11, 12]; self-efficacy to manage symptoms (COPD-Self Efficacy Scale) [13]; COPD knowledge (BCKQ) [14]; self-reported depression, fatigue, social support, and anxiety (PROMIS-forms) [15, 16]; Functional Capacity (TelePR: 6MWT (meters) and SPR: 2MST (steps), and perceived exercise capacity (measured via the Modified Borg Scale) [17–19].

##### Tertiary outcomes

We further assessed differences in uptake (participating in at least one PR session, which is described as ‘adoption’ in RE-AIM) and adherence (completion of the program and number of sessions attended of the 16-total PR sessions). In addition, we measured CAT, MMRC, PROMIS, and whether participants continued with exercise (yes/no self-report) at 6- and 12-months, and feasibility of equipment delivery and function in TelePR. We also recorded technical or other barriers to TelePR session completion, and participant satisfaction with the program (Table S1).

##### Sample-size and power calculation

The 276-person sample-size was based on an effect size of 0.3 odds ratio (OR), with 20% loss to follow-up and 80% power to detect superiority of referral to TelePR compared to SPR for the 6-month COPD readmission/mortality outcome using a two-sided chi-squared test at a significance level of 0.05 [10].

##### Quantitative analysis

There were three levels of analysis: Intention to Treat (ITT), which included all the people randomized at the beginning of the study (excluding those who were later found to not met inclusion criteria for referral, such as immobility after hospital discharge or PFT results that did not indicate COPD) (ITT); people randomly assigned to a study arm and medically cleared (Sub-analysis 1), and people who were randomly assigned, medically cleared, and who had participated in at least 1 PR session (Sub-analysis 2).

The ITT analysis compared outcomes for those randomized to TelePR vs. SPR, excluding those who would not have received a referral to PR in real-world practice (i.e., those who were later found not to meet inclusion criteria because they were immobile or did not have COPD, or who became medically unstable). We then performed 2 sub analyses: Sub-analysis 1 for patients who ultimately received medical clearances after referral (i.e., patients who would be allowed to *participate* in PR) and Sub-analysis 2 for patients who were medically cleared and then participated in at least 1 PR session.

Logistic regression analysis compared the odds ratio of the primary outcome, in 3 sets of models: *(Model 1)* intervention only with no other covariates added to the model (unadjusted); *(Model 2)* intervention, adjusted for race and clinical site (stratification variables); *(Model 3)* intervention, adjusted for race, clinical site, and risk factors shown to be associated with the primary outcome in the literature. We reviewed each study contained in the Cochrane Systematic Review [10, 20] and we identified 19 unique risk factors for COPD exacerbation admission: (1) depression [21, 22], (2) SES [23, 24], (3) heart disease [25], (4) male sex [22, 23], (5) nursing home residence [23], (6) age [21], (7) lower QOL [21], (8) prior hospitalization [26], (9) longer hospital length of stay [26], (10) higher number of comorbidities [24, 26], (11) need for long-term oxygen treatment [26], (12) poor lung functions [22, 24, 27], (13) marital status [24], (14) cor pulmonale [21, 28], (15) hypoproteinemia [28], (16) elevated PCO2 [28], (17) anemia [26], (18) low serum magnesium level [29], and (19) elevated C-reactive protein level [27]. Of the available patient data in our study, 6 of the risk factors (cor pulmonale, hypoproteinemia, elevated PCO_2_, anemia, low serum magnesium level, and elevated C-reactive protein level) were not reliable, because many people did not have these laboratory values in their EMR and so could not be included in the analyses. These analyses were performed for ITT group, Sub-analysis 1 and Sub-analysis 2. Therefore, there were a total of 9 analyses. We analyzed the data with a two-sided alpha = 0.05.

Continuous variables were summarized using mean, median and standard deviation; categorical variables were summarized using frequency and percentages. Two-sample t-test or nonparametric Wilcoxon test compared the continuous variables, and Chi-squared test or Fisher’s exact test compared categorical variables. To compare continuous variables between day 1 and 8-weeks, we used a paired t-test or nonparametric signed rank test, and a generalized linear mixed models (repeated measures analysis of variance “MMRMA”) to determine whether there was a difference in the change over time between the arms, and if the magnitude of change depended on treatment arm (treatment x time interaction). Unstructured covariance was used in all the models. Adherence was separately included as a covariable to examine its role on the primary outcome in the models.

##### Missing data

Missing data was handled using multiple imputation with details described in Appendix S1.

##### Qualitative methods and analysis

Qualitative interviews and focus groups conducted among a sample of participants allowed for a deeper understanding of (1) the barriers to initiating PR despite a referral to PR and (2) the barriers to participating in > 1 PR session once started (Appendix S2). Participants for the sub-study were recruited from among the 209 participants who had been enrolled into the wider study and represent those randomized to either TelePR or SPR. Interviews were conducted either in person or by phone by a member of the study team using the interview guides. Focus groups of participants were conducted in person by members of the study team using the focus-group guides. All interviews and focus groups were audio-recorded and transcribed by a professional medical transcribing company. Thematic analysis was performed by 3 members of the study team using the constant comparison method to create a codebook of themes with definitions, exemplary quotes, and exclusion and inclusion criteria. Transcripts were coded in NVivo Pro 12. A Kappa coefficient of less than 0.85 required discussion among the coders to resolve discrepancies and reach agreement.

#### Adoption

Adoption measures the proportion of people/settings who were willing to initiate the intervention. Because the intervention was in participants’ homes, the proportion of people who initiated TelePR sessions in their homes is the relevant metric for adoption and for describing barriers to adoption. In addition, we report the proportion of community centers that were willing to ‘house’ the TelePR sessions for participants who did not have space for equipment in their homes.

To increase adoption, we held meetings twice a year (estimated 10 meetings) with local clinicians informing them about the TelePR program - within the context of the clinical trial - including an email communication across the entire Northwell Health System by the chair of medicine, and presentations at division meetings. We worked with our CAB to identify methods to increase referral by hospital staff (physicians and respiratory therapists) for potentially eligible patients, and to increase adoption by the COPD patients and their families once approached by the study team. These meetings informed our recruitment materials targeted specifically to people from predominantly underserved Hispanic and African American people with COPD in the NYC metropolitan region. Details are provided in a separate manuscript [30].

#### Implementation

Implementation measures whether the intervention was delivered as intended. We include measurements of fidelity to clinical trial protocols, describing in detail any adaptations that were made based on CAB feedback as well as due to early findings in the clinical trial execution. We also report fidelity to the TelePR intervention components, to address specific factors relevant to TelePR sessions being executed as intended.

Our CAB provided recommendations for successful implementation of TelePR. This included improvements to equipment functionality, safety features for frail, older patients using the ergonomic stationary bike, providing a micro-key to the telehealth tablet computer to make it easier for older patients to turn it on and off and to access the features needed; and a laminated how-to sheet attached to the equipment. To increase retention among those patients who were enrolled in the program, we distributed a monthly newsletter, and a dedicated social worker helped participants obtain medical clearance appointments and associated considerations if they were re-hospitalized during the program.

#### Maintenance

Maintenance measures the continued use of the program over time. For this study, because it was funded by clinical trial grant monies, we measured maintenance on two levels. First, individual level maintenance of exercise by joining a gym or by continuing in a standard PR program. This does not reflect the value of TelePR directly but measures motivation that TelePR provided to exercise and the benefits of exercise. Second, we describe inquiries from health systems and pulmonary organizations to continue the TelePR programs, and the logistical and financial considerations that were discussed.

## Trial registration

The study was registered with the National Institutes of Health clinical trials registry. Study was registered on 02/01/2017 and registration number is NCT03007485. The trial registration website is www.clinicaltrials.gov.

## Results

### Reach

**Fig. 1:**
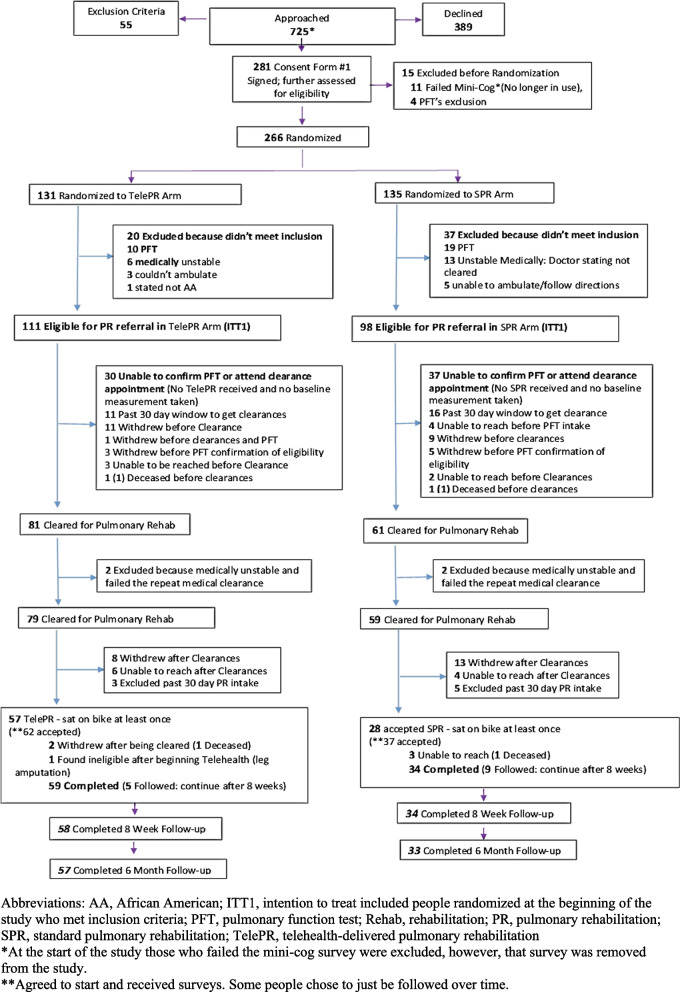
Consort Diagram – monitor and record participant recruitment, exclusion, and dropout. Abbreviations: AA, African American; ITT1, intention to treat included people randomized at the beginning of the study who met inclusion criteria; PFT, pulmonary function test; Rehab, rehabilitation; PR, pulmonary rehabilitation; SPR, standard pulmonary rehabilitation; TelePR, telehealth-delivered pulmonary rehabilitation. *At the start of the study those who failed the mini-cog survey were excluded, however, that survey was removed from the study. **Agreed to start and received surveys. Some people chose to just be followed over time

Recruitment occurred from April 2017 to June 2019. The trial was successful in recruiting from the intended patient population as outlined in Table 2. However, as seen in the CONSORT diagram, of the 725 potentially eligible people approached, only 281 were open to participation, 11 were excluded because of dementia and 4 were excluded because of PFT results excluded COPD. 266 people were randomized (131 TelePR; 135 SPR). Of the 131 randomized to TelePR, 20 were subsequently excluded after randomization because they longer met inclusion criteria (10 patients were ineligible due to PFT results indicating no COPD; 6 were too unstable medically to participate in PR, 3 because unable to ambulate or exercise, and 1 later stated they did not consider themselves African American). Of the 135 patients randomized to SPR, 37 were excluded after randomization because they no longer met inclusion criteria (19 patients were ineligible due to PFT results indicating no COPD; 13 became too unstable medically to participate in PR; 4 became unable to ambulate or exercise; and 1 could not follow directions required for exercise participation). In total, 209 participants were randomized and included in the ITT analysis (TelePR 111, SPR 98) (Fig. 1). Appendix S3 provides a detailed explanation of exclusions due to subsequent finding of ineligibility.

**Table 2:** Demographics for those who were medically cleared and participants who participated in PR sessions

	**TelePR**			**SPR**		
	**ITT** *N* = 111	**Medically Cleared (Subanalysis 1)** *N* = 79	**Participated in at Least 1 Session (Subanalysis 2)** *N* = 57	**ITT** *N* = 98)	**Medically Cleared (Subanalysis1)** *N* = 59	**Participated in at Least 1 Session (Subanalysis 2)** *N* = 28
**Gender, N (%)**						
Female	67 (60.36)	50 (63.29)	37 (64.91)	57 (58.16)	35 (59.32)	18 (64.29)
Male	44 (39.64)	29 (36.71)	20 (35.09)	41 (41.84)	24 (40.68)	10 (35.71)
**Age, categorical, N (%)**						
18–64	48 (43.24)	36 (45.57)	27 (47.37)	43 (43.88)	24 (40.68)	10 (35.71)
≥ 65	63 (56.76)	43 (54.43)	30 (52.63)	55 (56.12)	35 (59.32)	18 (64.29)
**Age, continuous, Mean (± SD)**						
	66.89 (10.80)	67.05 (10.64)	67.35 (12.05)	65.96 (10.68)	66.93 (9.74)	66.50 (9.96)
**FEV**_**1**_** (% predicted), Mean (± SD)**						
	51 (27) median: 47			48 (19) median: 46		
**Charlson Comorbidity Index Score, Mean (± SD)**						
	5.03 (2.29) median: 5.00	5.03 (2.41) median: 5.00	4.94 (2.44) median: 4.50	5.17 (2.58) median: 5.00	5.19 (2.60) median: 5.00	5.05 (2.59) median: 5.00
**Race/Ethnicity, N (%)**						
African American	63 (56.76)	46 (58.23)	31 (54.39)	57 (58.16)	35 (59.32)	16 (57.14)
Hispanic	48 (43.24)	33 (41.77)	26 (45.61)	41 (41.84)	24 (40.68)	12 (42.86)
**Insurance Status, N (%)**						
None	2.00 (1.80)	1.00 (1.27)	1.00 (1.75)	3.00 (3.06)	1.00 (1.69)	1.00 (3.57)
Medicaid	51.00 (45.95)	36.00 (45.57)	27.00 (47.37)	47.00 (47.96)	30.00 (50.85)	17.00 (60.71)
Medicare	60.00 (54.05)	45.00 (56.95)	31.00 (54.39)	45.00 (45.92)	28.00 (47.46)	15.00 (53.57)
Other	39.00 (35.14)	29.00 (36.71)	22.00 (38.60)	40.00 (40.82)	24.00 (40.68)	9.00 (32.14)
**Household Income, N (%)**						
Less than $49,00	67.00 (60.36)	50.00 (63.29)	36.00 (63.16)	68.00 (69.39)	43.00 (72.88)	21.00 (75.00)
$50–$99,000	7.00 (6.31)	4.00 (5.06)	2.00 (3.51)	7.00 (7.14)	5.00 (8.47)	3.00 (10.71)
$100,000–over	5.00 (4.50)	3.00 (3.80)	2.00 (3.51)	1.00 (1.02)		
Missing	32.00 (28.83)	22.00 (27.85)	17.00 (29.82)	22.00 (22.45)	11.00 (18.64)	4.00 (14.29)
**Self-Defined Social Economic Status, N (%)**						
Lower Class	24.00 (21.62)	17.00 (21.52)	14.00 (25.45)	32.00 (32.65)	18.00 (30.51)	9.00 (32.14)
Middle Class	58.00 (52.25)	41.00 (51.90)	28.00 (50.91)	38.00 (38.78)	24.00 (40.68)	11.00 (39.29)
Upper Class	1.00 (0.90)	1.00 (1.27)		1.00 (1.02)	1.00 (1.69)	1.00 (3.57)
Missing	28.00 (25.23)	20.00 (27.85)	13.00 (23.64)	27.00 (27.55)	16.00 (27.12)	7.00 (25.00)
**Educational Level, N (%)**						
Some High-School and less	31.00 (27.93)	22.00 (27.85)	19.00 (34.55)	25.00 (25.51)	14.00 (23.73)	6.00 (21.43)
High-School Graduate	36.00 (32.43)	24.00 (30.38)	17.00 (30.91)	33.00 (33.67)	19.00 (32.20)	7.00 (25.00)
Associate Degree and Higher	26.00 (23.42)	19.00 (24.05)	13.00 (23.64)	23.00 (23.47)	15.00 (25.42)	11.00 (39.29)
Missing	18.00 (16.22)	14.00 (17.72)	6.00 (10.91)	17.00 (17.35)	11.00 (18.64)	4.00 (14.29)
**Language Spoken Most Often, N (%)**						
English	72.00 (64.86)	51.00 (64.56)	36.00 (64.29)	62.00 (63.27)	39.00 (66.10)	18.00 (64.29)
Spanish	24.00 (21.62)	16.00 (20.25)			10.00 (16.95)	7.00 (25.00)
Both Languages Equally	7.00 (6.31)	6.00 (7.59)	5.00 (8.93)	12.00 (12.24)	6.00 (10.17)	3.00 (10.71)

### Effectiveness

#### Primary outcome

**Fig. 2:**
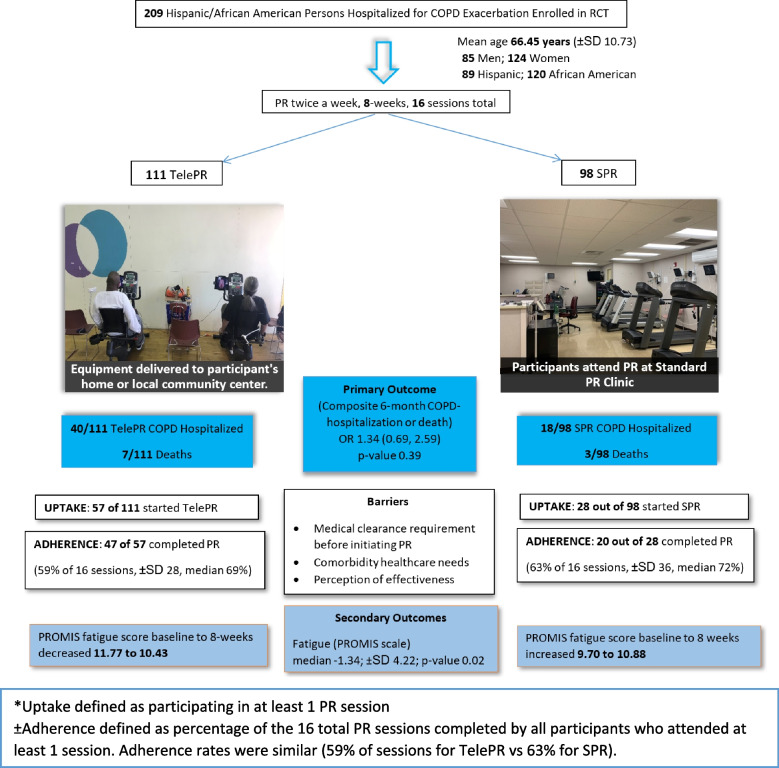
Overview of study including results for primary and secondary outcome and main barriers to PR

Although a total of 209 people were randomized to a referral TelePR vs. SPR (111 to TelePR and 98 to SPR), only 57 (51%) of the 111 people referred to TelePR, and 28 (28%) of the 98 referred to SPR, attended at least one PR session (referred to as ‘uptake’ and described further in the ‘adoption’ RE-AIM section below). A referral to TelePR did not appear to decrease the composite outcome of 6-month COPD readmission rate or death (OR1.35; 95% CI 0.69, 2.66) when compared to SPR. More specifically, within 6-months of enrollment, 40 out of 111 patients referred to TelePR (36%) were readmitted for COPD exacerbation, and 7 (6%) died; whereas 18 out of the 98 referred to SPR (18%) were readmitted and 3 died (3%) (Fig. 2).

None of the literature defined risk factors were associated with the outcomes in Model#3, therefore, results are identical to Model#2 (the model that adjusted for the stratification variables race and clinical site). Table 3 includes Model#1 (the unadjusted model) and #2 results for the three levels of analyses (ITT, Sub-analysis 1 and Sub-analysis 2). Tables 4 and 5 lists model results that include adherence as a covariable (i.e., binary variable; Table 4: > 75% of sessions attended, yes/no) and as a continuous variable (Table 5: percentage of sessions attended). These sub-analyses are very limited due to small sample sizes.

**Table 3:** Primary outcome – composite of COPD hospital readmission or death within 6 months of discharge using all available (complete) data, and no imputation of missing data

	**ITT** Odds ratio^a^ (TelePR vs. SPR) *N* = 209 (TelePR = 111, SPR = 98)	**Medically Cleared (Subanalysis 1)** Odds ratio^a^ (TelePR vs. SPR) *N* = 138 (TelePR = 79, SPR = 59)	**Participated in at Least 1 Session (Subanalysis 2)** Odds ratio^a^ (TelePR vs. SPR) *N* = 85 (TelePR = 57, SPR = 28)
Unadjusted (Model 1)	1.34 (0.69, 2.59) *p*-value = 0.3908	1.06 (0.43, 2.58) *p*-value = 0.9057	0.88 (0.29, 2.68) *p*-value = 0.8176
Adjusted (race, hospital) (Model 2)	1.35 (0.69, 2.66) *p*-value = 0.3837	1.09 (0.43, 2.73) *p*-value = 0.8578	0.86 (0.27, 2.72) *p*-value = 0.7930

(Model 1) Intervention only with no other covariates added to the model

(Model 2) Intervention, adjusted for race and clinical site (stratification variables)

(Model 3) not shown - intervention, race, clinical site, and literature reported risk factors for COPD-exacerbation hospitalization because no associations found between literatures reported risk factors and outcome, therefore identical to Model 2

^a^OR > 1.0 indicates that intervention increased incidence rate of composite outcome of death/hospital intervention

**Table 4:** Primary outcome with consideration of adherence > 75% (Yes/No) included as a covariable^a^ in models

	**ITT** Odds ratio ± (TelePR vs. SPR) *N* = 209 (TelePR = 111, SPR = 98)	**Medically Cleared (Subanalysis 1)** Odds ratio ± (TelePR vs. SPR) *N* = 138 (TelePR = 79, SPR = 59)	**Participated in at Least 1 Session (Subanalysis 2)** Odds ratio ± (TelePR vs. SPR) *N* = 85 (TelePR = 57, SPR = 28)
COPD Hospital Readmission			
Unadjusted (adherence > 75%) (model 1)	0.78 (0.28, 2.15) *p*-value = 0.6270	0.78 (0.28, 2.15) *p*-value = 0.6270	0.84 (0.27, 2.59) *p*-value = 0.7597
Adjusted (race, hospital, adherence > 75%) (model 2)	0.73 (0.25, 2.11) *p*-value = 0.5582	0.73 (0.25, 2.11) *p*-value = 0.5582	0.80 (0.25, 2.58) *p*-value = 0.7045

± OR > 1.0 indicates that intervention increased incidence rate of composite outcome of death/hospital intervention

^a^This analysis includes the percentages of the 16 sessions completed per person, from 0 to 100%

**Table 5:** Primary outcome with consideration of adherence as a percent of sessions attended^a^ included as a covariable in models

	**ITT** Odds ratio ± (TelePR vs. SPR) *N* = 209 (TelePR = 111, SPR = 98)	**Medically Cleared (Subanalysis 1)** Odds ratio ± (TelePR vs. SPR) *N* = 138 (TelePR = 79, SPR = 59)	**Participated in at Least 1 Session (Subanalysis 2)** Odds ratio ± (TelePR vs. SPR) *N* = 85 (TelePR = 57, SPR = 28)
COPD Hospital Readmission			
Unadjusted (adherence %) (model 1)	0.81 (0.29, 2.25) *p*-value = 0.6816	0.81 (0.29, 2.25) *p*-value = 0.6816	0.85 (0.28, 2.64) *p*-value = 0.7846
Adjusted (race, hospital, adherence %) (model 2)	0.76 (0.26, 2.19) *p*-value = 0.6059	0.76 (0.26, 2.19) *p*-value = 0.6059	0.81 (0.25, 2.61) *p*-value = 0.7208

± OR > 1.0 indicates that intervention increased incidence rate of composite outcome of death/hospital intervention

^a^This analysis includes the percentages of the 16 sessions completed per person, from 0 to 100%

#### Secondary outcomes

Subject to caution about interpreting secondary outcome *p*-values, and the small sample sizes of those who participated in PR, there were no statistically significant differences between the two arms for most of the secondary outcomes, except for the PROMIS fatigue score. This decreased on average (improved) in TelePR from day 1 to 8-weeks, whereas the fatigue score increased on average (worsened) in the SPR arm from day 1 to 8-weeks (Table 6).

**Table 6:** Outcomes before (day 1) and after (8 weeks) PR program completion for those who received medical clearance to participate

	**Trial arm (TelePR *n* = 62; SPR *n* = 37)**	**Day 1, mean (SD)**^**c**^**; median**	**8 Weeks, mean (SD); median**	**Change 8 weeks-Day 1, mean (SD)**^**c**^**, median**
COPD Assessment Test (CAT) Maximal Score: 40 (lower score denotes improvement)	TelePR (*n* = 62), Mean (± SD)	22.37 (8.35) median: 24.00	19.91 (7.94) median: 21.00	-2.27^a^ (8.43) median: -2, ***p* = 0.04**^†^
	SPR (*n* = 37), Mean (± SD)	20.89 (8.53) median: 24.00	20.18 (8.41) median: 20.00	-0.71^a^ (6.5) median: 0, *p* = 0.38^†^
	Effect size^b^			Effect size = 0.43
	*p*-value^§^	0.4031	0.9472	0.4201
	Overall *p*-value	ARM: 0.6977, Time < .0001, Time* ARM: 0.2855		
Modified Medical Research Council Scale (MMRC) Maximal Score: 4 (lower score denotes improvement)	TelePR (*n* = 62), Mean (± SD)	2.46 (1.16) median: 3.00	2.28 (1.14) median: 2.00	-0.15^a^ (1.01) median: 0, *p* = 0.25^†^
	SPR (*n* = 37), Mean (± SD)	2.24 (1.04) median: 2.00	2.24 (1.28) median: 2.00	0.00^a^ (1.37) median: 0, *p* = 1.00^†^
	Effect size^b^			Effect size = 0.12
	*p*-value^§^	0.2273	0.8551	0.4991
	Overall *p*-value	ARM: 0.8494, Time 0.8534, Time* ARM: 0.1719		
COPD Self-Efficacy Scale (CSES) Maximal Score: 170 (higher score denotes improvement)	TelePR (*n* = 62), Mean (± SD)	93.98 (27.70) median: 96.00	94.16 (29.30) median: 101.00	1.09^a^ (29.11) median: 2.50 *p* = 0.94^†^
	SPR (*n* = 37), Mean (± SD)	89.42 (33.42) median: 94.00	94.03 (28.59) median: 98.00	5.30^a^ (34.61) median: 0 *p* = 0.35^†^
	Effect size^b^			Effect size = 0.13
	*p*-value^§^	0.5361	0.8793	0.9871
Bristol COPD Knowledge Questionnaire (BCKQ) Maximal Score: 100 (higher score denotes improvement)	TelePR (*n* = 62), Mean (± SD)	30.35 (8.89) median: 32.00	34.03 (9.68) median: 36.50	4.19^a^ (8.51) median: 4.00 *p*** = 0.003**^†^
	SPR (*n* = 37), Mean (± SD)	30.81 (6.79) median: 30.00	32.33 (7.63) median: 33.00	1.79^a^ (7.74) median: 3.00 *p* = 0.17^†^
	Effect size^b^			Effect size = 0.30
	*p*-value^§^	0.9142	0.2391	0.2311
PROMIS: Depression Maximal Score: 20 (lower score denotes improvement)	TelePR (*n* = 62), Mean (± SD)	8.35 (4.21) median: 8.00 T-score: 55.7 (2.3)	7.97 (4.48) median: 6.50 T-score: 55.7 (2.3)	-0.32^a^ (3.42) median: 0 *p* = 0.46^†^
	SPR (*n* = 37), Mean (± SD)	7.38 (4.24) median: 6.00 T-score: 53.9 (2.4)	7.85 (3.78) median: 7.00 T-score: 55.7 (2.3)	0.52^a^ (3.96) median: 0, *p* = 0.46^†^
	Effect size^b^			Effect size = 0.23
	p-value^§^	0.2533	0.8361	0.4087
	Overall p-value	ARM: 0.9561, Time 0.1274, Time* ARM: 0.3290		
PROMIS: Fatigue Maximal Score: 20 (lower score denotes improvement)	TelePR (*n* = 62), Mean (± SD)	11.77 (4.95) median: 10.50 T-score: 57.0 (2.3)	10.43 (4.53) median: 10.50 T-score: 53.1 (2.4)	-1.34^a^ (4.22) median: -1 ***p* = 0.02**^†^
	SPR (*n* = 37), Mean (± SD)	9.70 (4.47) median: 9.00 T-score: 53.1 (2.4)	10.88 (4.21) median: 11.00 T-score:55.1 (2.4)	1.45^a^ (5.41) median: 1, *p* = 0.13^†^
	Effect size^b^			Effect size = 0.58
	*p*-value^§^	0.0707	0.5933	0.0071
	Overall *p*-value^ll^	ARM: 0.6812, Time 0.5964, Time* ARM: 0.0380		
PROMIS: Informational Support Maximal Score: 20 (lower score denotes improvement)	TelePR (*n* = 62), Mean (± SD)	14.95 (5.24) median: 17.50 T-score: 50.3 (2.4)	16.10 (4.61) median: 17.00 T-score: 52.4 (2.4)	1.00^a^ (5.38) median: 0 *p* = 0.16^†^
	SPR (*n* = 37), Mean (± SD)	15.76 (4.79) median: 16.00 T-score: 52.4 (2.4)	15.64 (4.70) median: 17.00 T-score: 52.4 (2.4)	-0.15^a^ (4.37) median: 0, *p* = 0.84^†^
	Effect size^b^			Effect size = 0.17
	*p*-value^§^	0.4224	0.5797	0.6808
	Overall *p*-value ^ll^	ARM: 0.3643, Time 0.4149, Time* ARM: 0.4772		
PROMIS: Social Isolation Maximal Score: 20 (lower score denotes improvement)	TelePR (*n* = 62), Mean (± SD)	7.97 (4.34) median: 6.00 T-score: 47.8 (2.6)	8.66 (4.56) median: 7.00 T-score: 49.8 (2.6)	1.03^a^ (3.60) median: 0 *p*** = 0.03**^†^
	SPR (*n* = 37), Mean (± SD)	7.39 (3.77) median: 7.00 T-score: 45.7 (2.7)	8.79 (4.54) median: 8.00 49.8 (2.6)	1.39^a^ (5.06) median: 1, *p* = 0.12^†^
	Effect size^b^			Effect size = 0.08
	*p*-value^§^	0.7009	0.7768	0.583
	Overall p-value ^ll^	ARM: 0.8534, Time 0.0622, Time*ARM: 0.3248		
PROMIS: Instrumental Support Maximal Score: 20 (lower score denotes improvement)	TelePR (*n* = 62), Mean (± SD)	15.60 (4.90) median: 17.50 T-score: 50.5 (2.4)	15.53 (5.01) median: 17.00 T-score: 50.5 (2.4)	-0.22^a^ (3.12) median: 0 *p* = 0.70^†^
	SPR (*n* = 37), Mean (± SD)	15.70 (5.24) median: 17.00 T-score: 50.5 (2.4)	16.00 (4.37) median: 18.00 T-score: 50.5 (2.4)	0.67^a^ (4.81) median: 0, *p* = 0.53^†^
	Effect size^b^			Effect size = 0.22
	*p*-value^§^	0.8612	0.9628	0.65
	Overall *p*-value ^ll^	ARM: 0.8475, Time 0.9577, Time* ARM: 0.6395		
PROMIS: Anxiety Maximal Score: 20 (lower score denotes improvement)	TelePR (*n* = 62), Mean (± SD)	8.71 (4.21) median: 8.00 T-score: 57.7 (2.6)	8.29 (4.40) median: 7.00 T-score: 55.8 (2.7)	-0.52^a^ (3.30), median: 0 *p* = 0.38^†^
	SPR (*n* = 37), Mean (± SD)	8.38 (4.70) median: 8.00 T-score: 55.8 (2.7)	8.85 (4.14) median: 8.00 T-score: 57.7 (2.6)	0.33^a^ (3.89) median: 0, *p* = 0.84^†^
	Effect size^b^			Effect size = 0.24
	*p*-value^§^	0.6369	0.3217	0.86
	Overall *p*-value ^ll^	ARM: 0.7752, Time 0.9675, Time* ARM: 0.7143		
PROMIS: Companionship Maximal Score: 20 (lower score denotes improvement)	TelePR (*n* = 62), Mean (± SD)	14.26 (4.76) median: 14.00 T-score: 46.2 (2.2)	14.88 (4.68) median: 16.00 T-score: 48.1 (2.2)	0.74^a^ (3.58) median: 0 *p* = 0.21^†^
	SPR (*n* = 37), Mean (± SD)	14.14 (4.93) median: 14.00 T-score: 46.2 (2.2)	13.27 (4.42) median: 14.00 T-score: 44.3 (2.2	-0.33^a^ (5.21) median: 0, *p* = 0.84^†^
	Effect size^b^			Effect size = 0.24
	*p*-value^§^	0.9275	0.073	0.41
	Overall p-value ^ll^	ARM: 0.9979, Time 0.5391, Time* ARM: 0.1219		
Functional Capacity (TelePR: 2MST (steps); SPR: 6MWT (meters)) (higher denotes improvement)	TelePR (*n* = 62), Mean (± SD)	43.87 (17.42) median: 42.00	51.12 (23.46) median: 50.00	1.22^a^ (± 29.5%) median: 1.22 *p* = 0.001^**^
	SPR (*n* = 37), Mean (± SD)	261.92 (125.85) median: 269.00	275.29 (137.85) median: 320.00	0.98^a^ (± 130%) median: 1.12 *p* = 0.911^**^
	Effect size^b^			Effect size = 0.003
	*p*-value^§^			0.22

All *p*-values are for descriptive purposes and should be interpreted with caution

*Abbreviations*: *2MST* 2 min-step test, *6MWT* 6 min-walk test

^†^Paired t-test or signed rank test

^§^Two-sample t-test or Wilcoxon rank sum test

^ll^*p*-values for each variable from repeated measures analysis of variance “MMRMA” (*p*-value for Arm: if there was difference between two arms in the outcome variable, *p*-value for Time: if the outcome changed from 8 weeks to day 1; *p*-value for Arm*Time: if the changes of the outcome variable over time differ between the two arms. For example, for CAT, no difference in CAT between the two arms, a significant improvement in CAT over time, but the changes over time did not differ between the two arms

^**^Paired t-test using log transformation

^a^Change in 8 weeks-Day1: negative values denote improvement; positive values denote worsening; Geometric mean of the ratio 8-week score to day 1 score; larger values indicate improvement

^b^Effect Size: Cohen’s D for two-sample t-test = difference in the mean/pooled standard deviation

^c^Functional capacity was analyzed as percent change from baseline to 8 weeks. The data was analyzed using a log transformation of the data and results are presented as geometric means and geometric standard deviations. This analysis is based on the log-fold change from baseline to 8 weeks in number of steps (for 2 min walk test). 1.22 represents the geometric mean fold change from baseline (i.e., a 22% increase). ± 29.5% allows us to say that 95% of the *population* would lie between a 0.16 fold change and a 9.46 fold change, where the 0.16 represents an 84% decrease in distance walked in 6 min and 9.46 represents an 846% increase in distance. For the SPR group the same interpretation applies where 0.98 represents the geometric mean fold change from baseline (i.e., a 2% decrease). ± 130% allows us to say that 95% of the *population* would lie between a 0.6 fold change and a 1.59 fold change where the 0.6 represents a 40% decrease in steps and 9.46 represents a 59% increase in steps climbed in 2 min

When comparisons were made within each arm, TelePR participants experienced improvements in the following outcomes from baseline to 8-weeks: COPD symptoms (CAT and MMRC); knowledge about COPD management (BCKQ); fatigue, and functional capacity. These improvements were not seen among those in SPR. With increased exercise tolerance and training, it would be expected that before and after exercise (when the BORG was measured), the difference in perceived dyspnea (measured using the Modified Borg Scale) would be less, suggesting improved endurance. An improvement in the BORG scale seen among SPR participants from day 1 to 8 weeks (Table S2), which was not seen among those in TelePR. However, even at baseline (ie, before starting PR), it appeared that those in the TelePR arm were more deconditioned, as suggested by greater dyspnea with exercise after the session. Nevertheless, TelePR had a statistically significant difference in the increase in exercise resistance level (i.e., resistance number on the bike able to be tolerated by the participant) over time from baseline to 8 weeks compared to SPR participants (Fig. S4), and there was a greater mean duration of exercise which may have explained the increased perceived exertion measured by the BORG scale (Fig. S5).

The objective measurement of improvement in exercise capacity would have been a comparison in the 6MWT measure. However, due to space constraints in participants homes, and concerns about gait stability, we could not obtain 6MWT results and instead used the 2MST for the TelePR arm which allows measurement while participants stands in place and lifts their legs to hip height consecutively vs walking in a flat distance of at least 25 m [17]. Changes in functional capacity score (assessed with the 6MWT and 2MST) could therefore not be compared between arms. Participants in TelePR did experience an improvement in the 2MST from day 1 to 8-weeks.

#### Tertiary outcomes

Most patients who started PR completed the program, with 47 of 57 (82%) participants in TelePR, and 20 of 28 (71%) participants in SPR completing the program. The mean number of PR sessions attended out of the 16-total was similar in both arms (TelePR 59.21% (± SD 27.85), median 68.75%; and SPR 62.95% (± SD 35.76), median: 71.88%).

#### Longitudinal changes over one-year follow-up

Improvements seen from baseline to 8-weeks (i.e., from before to after completing the 8-week PR program) did not persist at 12-months of follow-up. All secondary outcomes (i.e., CAT, MMRC, and PROMIS) regressed to baseline scores. For example, at baseline, TelePR had a 22.27(8.35); 24.00 (mean (SD; median) and SPR had a 20.89(8.53); 24.00 for the CAT score. During 12-month assessment, TelePR had a 23.00(8.26); 23.00 and SPR had a 20.09(7.70); 21.500 for the CAT score -demonstrating that the scores had regressed. Very few participants (22.50% TelePR and 12.50% SPR) reported that they were continuing to exercise (Table S3).

### Adoption

Of the 111 people referred to TelePR only 57 (51%) attended at least one PR session. In comparison, only 28 (28%) of the 98 referred to SPR attended at least one PR session. Therefore, although adoption was low, a higher proportion of patients were willing to initiate TelePR than SPR. There were several barriers to community settings agreeing to have the TelePR sessions conducted from their sites which included concerns about equipment theft, climate control for storage, and safety concerns in case other people used the equipment. There were also liability concerns that needed to be addressed by our legal consultants.

Our qualitative interviews revealed that one important barrier to TelePR adoption was related to beliefs that PR itself was not effective because patients’ clinicians had not previously recommended it. An illustrative quote is: “… *my doctor never told me anything about that [PR]. I mean, he could tell his patients when they have severe – chronic breathing, respiratory infections, and things like that. He should advise his patients to just give it a try. If he would have told me to go, I would have went.”* On the other hand, many potential participants expressed a “catch 22” of being too sick to exercise even though they knew it could be beneficial.

Barriers to participation in any form of PR included having to prioritize treating more pressing health concerns such as cancer or dialysis appointments. Comorbidities prevented adoption due to scheduling conflict concerns with other clinic appointments, and concern about burden to family members who would be needed for transportation and for attending sessions. In fact, once a referral was made to either TelePR or SPR, the need to attend an in-person medical clearance exam was a common barrier to completing the referral. Social constraints included caring for grandchildren and the need to have someone in the home to help them start the video sessions for TelePR. Further, some patients did not have adequate space in the home to store the equipment or did not have a permanent residence but did not want to attend sessions in community centers due to lack of transportation or feeling too ill to leave the home.

### Implementation

Overall, the TelePR program was able to be successfully implemented.

#### Fidelity to clinical trial protocol and adaptations made

Adaptations that needed to be made to the protocol included the addition of a qualitative component to understand why we had difficulty enrolling patients. Thirty-nine qualitative interviews were conducted among participants who either did not complete the PR program (*n* = 19) or did complete 8 weeks of either TelePR or SPR (*n* = 20) to determine factors impacting adoption and adherence to the PR (Table S4). Of those 39, 28 participants had the interviews within the year that they either completed, were excluded, or withdrew from the PR program, 8 participants had the interviews between a year and 18-months after completing, being excluded or withdrawing from the PR program, and 3 participants had the interviews 2 years after being excluded and withdrawing from the PR program. Interviews ranged between 30 min to 1 h. Two focus group with 10 participants (5 TelePR and 5 SPR) were subsequently conducted among those interviewed.

#### Fidelity to components of the TelePR program

The TelePR program to be tested in the RCT was a new program developed by the investigators. Therefore, the equipment used required usability testing to support user-centered design before enrolling patients. We studied barriers to implementation of the intervention while the clinical trial was ongoing and made necessary adaptations to equipment and enrollment materials in real-time. These were all recorded and are reported below.

Of the 52 participants who chose to receive TelePR in their own homes, 47 completed the full 8 weeks. On average participants completed a median of 11 sessions out of 16 total sessions with most sessions missed due to comorbidity related clinic visits or hospitalization (Table 7).

**Table 7:** PR adherence for TelePR and SPR who participated in at least one PR session

	Sessions completed out of 16 sessions Mean (± SD)	Average % of sessions completed Mean % (± SD)
**TelePR *n* = 57**	9.47 (4.46) median: 11.00	59.21% (27.85 Median: 68.75%
**SPR *n* = 28**	10.07 (5.72) median: 11.50	62.95% (35.76) Median: 71.88
*P*-value	0.3778	0.3778

##### Usability testing for the TelePR platform

We performed in-lab and in-field usability testing to check interactions with the TelePR equipment and make necessary refinements [31].

Adaptations included: having the RT review a checklist with participants (i.e., water, oxygen, if necessary, weights, band, and a phone in case of emergency), adjusting the tablet mounting on the bike, making the icons on the tablet larger, and for those patients unable to complete TelePR sessions without in-person support – having either a caregiver or study team member present during the PR session.

##### Delivery feasibility and equipment function in TelePR

All the bikes were able to be delivered to TelePR participants’ homes, with only minor issues that were easily addressed. Home set-ups were almost all without problem. Minor delays included: bike malfunctions, technical difficulties with the tablet computer, internet connection issues, and for those not having an AC during the summer inability to exercise. Overall, the main barriers faced during sessions were technical difficulties with the software due to internet speed, and difficulty interacting with the equipment (tablet computer, launching the software, and adjusting the bike) that were addressed in usability testing. These adaptations are reported in detail separately [31]. All technical difficulties were remedied remotely via phone support and screen-sharing. However, 15 participants out of 57 who participated in TelePR sessions needed the staff to travel to their home or community center to assist with difficulties for the first session.

The staffing necessary to execute the TelePR program included a bilingual respiratory therapist and social worker, pulmonologist, recruiters, clinical research coordinators, and a study manager. After the trial was completed, there was strong support by the department of medicine, as well as by the patients who had completed the program to continue offering TelePR.

### Maintenance

For our program, maintenance covers two distinct areas: 1) among the patients who completed the program, the adoption of long-term behavior changes learned though PR participation including regular exercise, and breathing techniques; and 2) among providers, the extent to which PR clinics have incorporated TelePR into their standard services for their chronic pulmonary patients in the post-study period.

Only 5 of the 57 people in TelePR continued some form of exercise at 6 months after study enrollment. Several participants stated that they felt the program had been taken away from them, almost as if a ‘rug had been pulled out from beneath them’ despite efforts by the study team to inform them of local gyms for continued exercise and the delivery of the stationary peddler for continued exercise. In fact, at the end of the program and compared to the start of PR for both TelePR and SPR participants, the social isolation score worsened likely reflecting this feeling of being abandoned after such frequent contact with the PR group. Several participants stated they were continuing to engage in some form of exercise (e.g., walking). Unfortunately, for many patients who had benefitted from PR, continued access to a PR program once their time in the study had ended was not possible due to transportation and financial difficulties and long waitlists for PR.

Among providers, during the COVID-9 pandemic, rapid changes in telehealth permissible platforms and insurance reimbursement structures occurred. The Pulmonary division at the largest hospital adopted the same tools that the trial used for vital sign monitoring, and the protocol for TelePR sessions, using a different web-based platform. Unfortunately, nation-wide staffing shortages of respiratory therapists prevented this program from being implemented. Very low rates of insurance reimbursement for PR and for telehealth visits are further barriers. This demonstrates that TelePR is not anymore of a barrier compared to SPR.

## Discussion

TelePR is equivalent to SPR in terms of QOL and exercise capacity, and superior in terms of patient adherence to PR. Stickland et al. [32] demonstrated in their study that TelePR was effective in increasing PR for individuals with COPD, as patients participating in PR showed improved QOL and exercise capacity comparable with those participating in SPR. Paneroni et al. [33] demonstrated that participants in a TelePR program had improved walking capacity, dyspnea, QOL, and daily physical activity. In addition, Holland et al. [34] provided evidence that TelePR was safe and feasible for individuals with COPD. TelePR has the potential to overcome many barriers that are more pronounced in patients with health disparities. Unfortunately, there have been very few studies comparing the effectiveness of TelePR with SPR in populations of patients at increased risk of health and health care disparities. We undertook a study to compare the effectiveness of a referral to TelePR vs SPR in patients discharged for an acute exacerbation of COPD.

Using the RE-AIM framework we demonstrate that TelePR was able to reach the intended population; was not comparatively superior to those referred to SPR; was effective in improving health outcomes when evaluating pre-and post- health metrics for those who completed TelePR including improved COPD knowledge and management skills, decreased fatigue and improved functional capacity; had low rates of adoption due to perceived benefit of exercise and social challenges largely related to comorbidities and needing in-person medical clearance appointments – although there was higher adoption compared to those referred to SPR; was able to be successfully implemented in patients’ homes; and had low maintenance rates after the program was discontinued. Several participants stated they were continuing to engage in some form of exercise (eg, walking) but those who were no longer engaging in physical activity cited during the interviews that a lack of access either to a PR facility or to the exercise equipment as barriers to continued physical activity.

It is possible to successfully conduct TelePR sessions for people with advanced stage COPD with multiple comorbidities who were recently hospitalized, and for people who are from typically underserved Spanish-only speaking and/or Black communities. Many TelePR participants stated that watching other patients with similar health problems as themselves via teleconference motived them to push themselves to stay on the same exercise level or, at times, be better. Because of the various comorbidities that individuals with COPD face, social isolation is a common factor [35, 36]. TelePR allowed participants to build friendships and find a connection with each other. Additionally, both groups included feeling too sick to exercise and having to prioritize other comorbidities over PR (e.g., cancer treatment, dialysis) as primary barriers to participation. Although TelePR removed the transportation barriers to receiving PR by making it possible to do PR in patients’ homes, many participants still had the transportation barrier of getting to appointments for medical clearances. Comorbidities interfered with participation in exercise sessions due to the need to attend medical appointments at the same time. We have detailed the myriad considerations necessary for successfully implementing a program such as ours and the critical importance of early usability testing and of community engagement via our CAB [30].

We identified several barriers to adoption of TelePR by patients, the most significant of which was beliefs about the importance of exercise compared to other types of disease management such as medication. This sometimes led to deliberative and informed decisions not to take part in PR despite the support of a social worker and study team. Our recruitment videos attempted to address beliefs about exercise futility using patient-testimonials. However, effective messaging must come from multiple outlets to influence social norms about exercise in advanced stage lung disease. This includes buy-in from primary care physicians about the feasibility and safety of PR for these patients.

For those who accepted a referral to TelePR, the most significant barrier to starting was the requirement for in-person medical clearance. It is possible that with the increasing prevalence of telehealth visits, preliminary clearance could be obtained before hospital discharge, with a follow up via telehealth prior to initiating the first TelePR session. Several hospitalists, primary care and even some pulmonary clinicians were reluctant to ‘clear’ patients with such advanced lung disease for exercise, fearing adverse events during exercise. The same reluctance was not encountered among cardiologists. This may be an area of necessary education regarding the relative benefit of exercise therapy and the other components of PR – education and socialization- that outweigh harms. In fact, there were no serious adverse events related to PR in our study.

A particularly important lesson learned is the lack of maintenance of exercise once people completed the 8-week program and the clear necessity for maintenance PR to be available for people with advanced stage COPD to prolong the achieved health improvements. Unfortunately, improvements seen in secondary outcomes did not persist at 6 and 12 months, which was not unexpected, because few, if any, patients had access to PR beyond the research study. This is a known limitation of PR programs, wherein, due to insurance reimbursement constraints and long wait lists for new PR participants, there is a limited number of PR sessions available to patients. Although preparation to transition out of the PR program includes conversations about the importance of maintaining exercise to preserve the gains made during the program, many patients do not continue exercise, as evidenced by the follow-up phone calls. Participants frequently stated they would have liked to continue attending the PR sessions (both TelePR and SPR) but that they were unable to do so due to transportation and financial difficulties. Patients in real-world PR programs are invited to continue in a maintenance program such as once-weekly sessions; however, there are long waitlists and one main barrier to PR maintenance is that many insurance policies limit the number of PR sessions to 72 sessions within a lifetime, if they cover it at all – having people pay out of pocket. Prior studies have shown significant benefit while in a PR maintenance program for 4–5 years compared to short-term PR in individuals with COPD [37]. With TelePR a tailored de-escalation of the number of supervised sessions could be performed with ‘booster’ sessions as needed over time to maintain health benefits.

In summary, while we were able to reach our intended target population and to implement a TelePR intervention that maintained high fidelity to standard PR, we faced multiple barriers to adoption and maintenance at both patient and provider levels. Therefore, we recommend increased messaging from primary care providers and community-based outlets about the benefits of PR, facilitating referrals and medical clearances for PR, supporting wide-scale TelePR availability, and supporting reimbursement for maintenance PR programs.

### Study limitations

The most important study limitation is the small sample size of 85 participants who completed the referral and attended at least one PR session in either arm of the study. This limits our ability to conclude whether participation in TelePR (as opposed to a referral to TelePR) decreases 6-month readmissions/death when compared to those participating in SPR. An additional limitation is that we applied the RE-AIM framework in a *post-hoc* analysis. Therefore, our understanding of barriers to adoption and retention are limited to what participants who were interviewed chose to share with us and these were not systematically collected from all participants. Nevertheless, several of the parameters needed for RE-AIM were included in the original RCT protocol and were prospectively collected such as adherence and retention rates, maintenance of exercise and target population demographics.

## Conclusion

TelePR is safe and effective to implement among people with advanced stage COPD and comorbidities from underserved Hispanic and Black communities. Although facilitating access to PR allowed us to control for common socioeconomic factors that typically prevent populations experiencing health/health care disparities from accessing PR, beliefs about the utility and safety of exercise therapy, and the burden of competing comorbidities are barriers to adoption. Messaging to clinicians and communities about the benefits of PR and messaging to payors about potential cost-savings of subsidizing PR and PR maintenance is necessary for improved adoption and for dissemination.

## Supplementary Information

**Additional file 1: Figure S1.** Participant flow in the study. **Figure S2.** Consenting process during formal study. Participants were requested to sign consent form 1. For those randomized to TelePR they were requested to sign consent form 2. **Figure S3.** Overview of study equipment for the TelePR intervention. **Table S1.** Satisfaction Survey results for TelePR (*n* = 57) and SPR (*n* = 28) participants at the time of completion of PR (8 weeks). **Appendix S1. Table S2.** Modified Borg Scale administered to participants in both TelePR and SPR at Day 1 (session 1) and 8-weeks (session 16). **Figure S4.** Resistance Level on Bicycle (TelePR) and treadmill (SPR) from Day 1 (start of PR) to Day 16 (completion of PR). **Figure S5.** Duration on Bicycle (TelePR) and treadmill (SPR) from Day 1 (start of PR) to Day 16 (completion of PR). **Table S3.** Longitudinal Outcomes. **Appendix S2.** Overview of the Interviews and focus groups pre- and post- RCT. **Table S4.** Focus group and interviewed participant’s demographics. **Appendix S3.**

## Data Availability

All data generated or analyzed during this study are included in this article (and its supplementary information files).

## Abbreviations

COPD: Chronic obstructive pulmonary disease
PR: Pulmonary rehabilitation
TelePR: Telehealth-delivered pulmonary rehabilitation
SPR: Standard pulmonary rehabilitation
MZRCF: Modified Zelen randomized consent form
PFT: Pulmonary function test
RT: Respiratory therapist
CAB: Community advisory board
QOL: Quality of life
CAT: COPD Assessment Test
MMRC: Modified Medical Research Council
BCKQ: Bristol COPD Knowledge Questionnaire
OR: Odds ratio
ITT: Intention to treat
BORG: BORG Ration Scale of Perceived Exertion
6MWT: 6-Minute walk test
2MST: 2-Minute step test
AC: Air conditioner
COVID-19: Coronavirus Disease 2019
