# Supplementary material for: Application of the RE-AIM framework to evaluate the implementation of telehealth pulmonary rehabilitation in a randomized controlled trial among African-American and Hispanic patients with advanced stage Chronic Obstructive Pulmonary Disease
Source: BMC Health Serv Res. 2023 May 23;23:515. doi: 10.1186/s12913-023-09492-7 (PMC10202528; doi:10.1186/s12913-023-09492-7)
Supplement: Supplementary file 1 — Additional file 1: Figure S1. Participant flow in the study. Figure S2. Consenting process during formal study. Participants were requested to sign consent form 1. For those randomized to TelePR they were requested to sign consent form 2. Figure S3. Overview of study equipment for the TelePR intervention. Table S1. Satisfaction Survey results for TelePR (n = 57) and SPR (n = 28) participants at the time of completion of PR (8 weeks). Appendix S1. Table S2. Modified Borg Scale administered to participants in both TelePR and SPR at Day 1 (session 1) and 8-weeks (session 16). Figure S4. Resistance Level on Bicycle (TelePR) and treadmill (SPR) from Day 1 (start of PR) to Day 16 (completion of PR). Figure S5. Duration on Bicycle (TelePR) and treadmill (SPR) from Day 1 (start of PR) to Day 16 (completion of PR). Table S3. Longitudinal Outcomes. Appendix S2. Overview of the Interviews and focus groups pre- and post- RCT. Table S4. Focus group and interviewed participant’s demographics. Appendix S3. [file 12913_2023_9492_MOESM1_ESM.docx]

**Title:** Application of the RE-AIM framework to evaluate the implementation of telehealth pulmonary rehabilitation in a randomized controlled trial among African American and Hispanic patients with advanced stage COPD.

**Authors:** Jennifer Polo, BA, Meng Zhang, PhD, Melissa J Basile, PhD, Keyla Ordonez, BS, Danielle Rodriguez, MHA, Eugenia, Boye-Codjoe, MPH, Myia Williams, PhD, Donna Tsang, RRT, CPFT, Richard Medina, RRT, Sonia Jacome, MSCH, Parvez Mir, MD, Sameer Khanijo, MD, Renee Pekmezaris, PhD, Negin Hajizadeh, MD, MPH

**Supplementary Material**

**Figure S1.** Participant flow in the study.


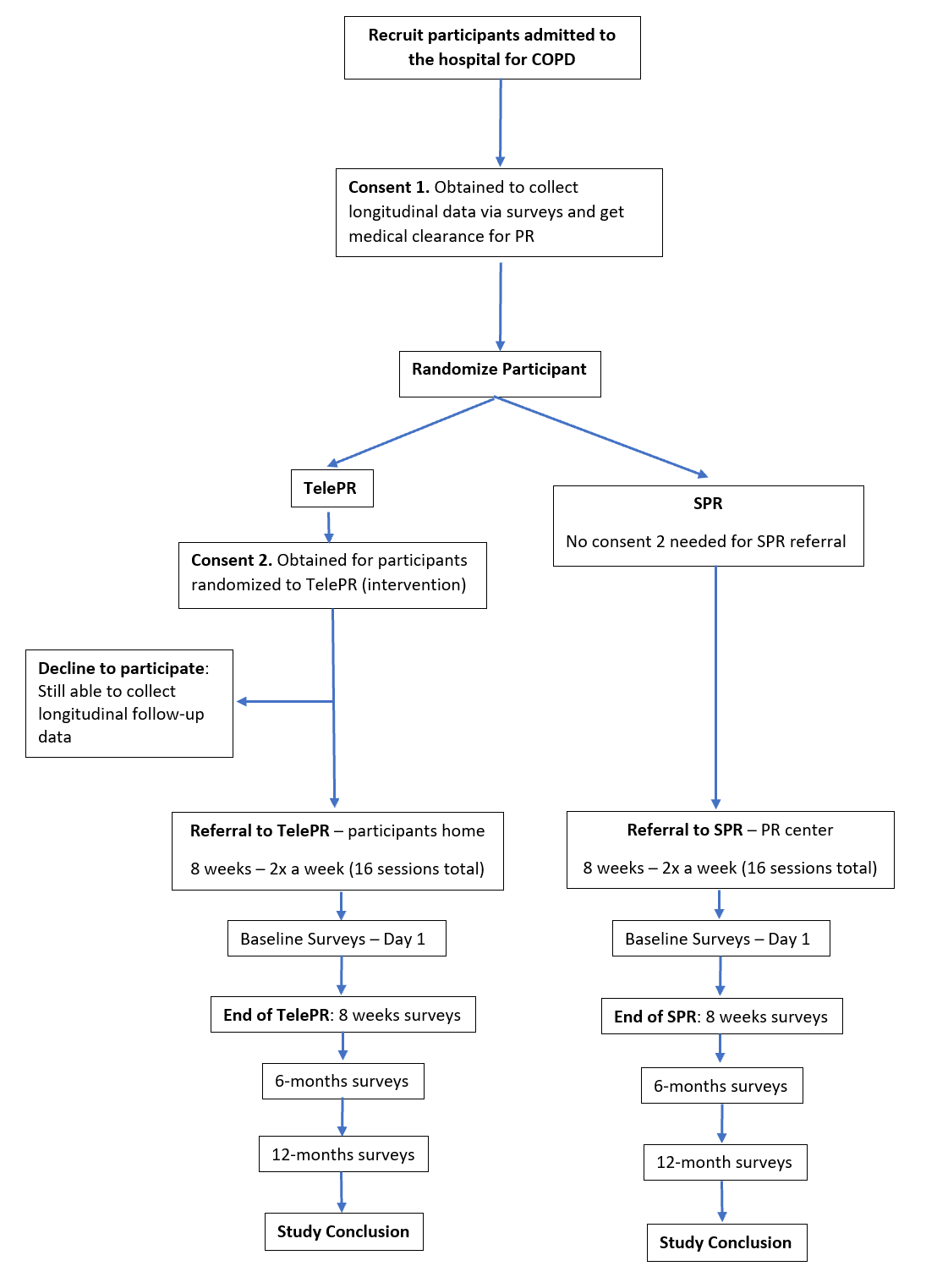


**Figure S2.** Consenting process during formal study. Participants were requested to sign consent form 1. For those randomized to TelePR they were requested to sign consent form 2.


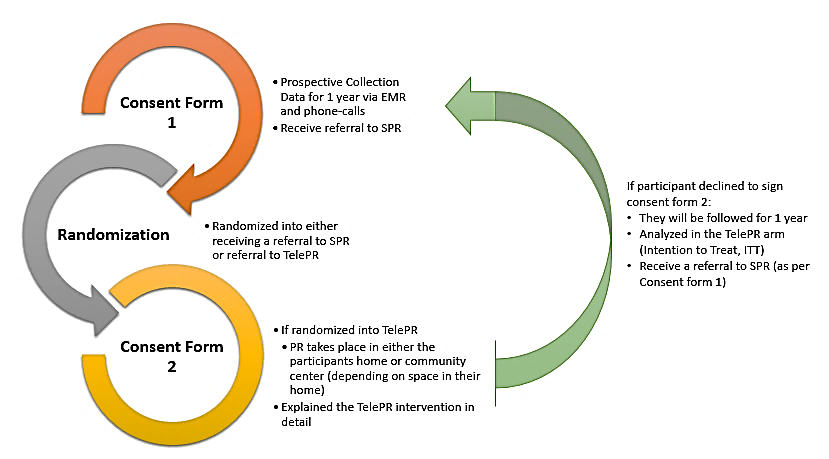


**Figure S3.** Overview of study equipment for the TelePR intervention.


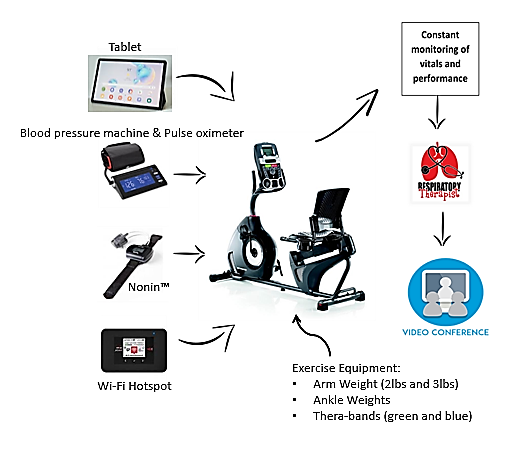


**Table S1.** Satisfaction Survey results for TelePR (n=57) and SPR (n=28) participants at the time of completion of PR (8 weeks).

| **Satisfaction Survey Questions** | **PR ARMS** | **Very Satisfied**  n (%) | **Somewhat Satisfied**  n (%) | **Neither Satisfied nor Dissatisfied**  n (%) | **Somewhat Dissatisfied**  n (%) | **Very Dissatisfied**  n (%) | **p-value** |
| --- | --- | --- | --- | --- | --- | --- | --- |
| **Overall how satisfied or dissatisfied are you with PR program?** | TelePR | 47.00 (85.45) | 6.00 (10.91) | 1.00 (1.82) | 1.00 (1.82) |  | 0.8776 |
|  | SPR | 23.00 (85.19) | 4.00 (14.81) | 0.00 (0.00) | 0.00 (0.00) |  |  |
| **How successful or unsuccessful do you feel the PR program has been in improving your health?** | TelePR | 37.00 (67.27) | 13.00 (23.64) | 4.00 (7.27) | 1.00 (1.82) |  | 0.6904 |
|  | SPR | 22.00 (81.48) | 4.00 (14.81) | 1.00 (3.70) | 0.00 (0.00) |  |  |
| **How easy or difficult was it for you to communicate with the PR team?** | TelePR | 52.00 (92.86) | 4.00 (7.14) |  | 0.00 (0.00) |  | 0.1803 |
|  | SPR | 23.00 (85.19) | 2.00 (7.41) |  | 2.00 (7.41) |  |  |
| **How helpful or unhelpful was the PR team?** | TelePR | 52.00 (92.86) | 4.00 (7.14) | 0.00 (0.00) |  |  | 0.0443 |
|  | SPR | 22.00 (81.48) | 2.00 (7.14) | 3.00 (11.11) |  |  |  |
| **How easy or difficult was it to learn to use the exercise bike?** | TelePR | 34.00 (64.15) | 13.00 (24.53) | 1.00 (1.89) | 3.00 (5.66) | 2.00 (3.77) | 0.1150 |
|  | SPR | 19.00 (76.00) | 2.00 (8.00) | 3.00 (12.00) | 1.00 (4.00) | 0.00 (0.00) |  |
| **How easy or difficult was it to use the equipment to measure your vital signs?** | TelePR | 41.00 (75.93) | 9.00 (16.67) | 1.00 (1.85) | 3.00 (5.56) |  | 0.5803 |
|  | SPR | 18.00 (69.23) | 5.00 (19.23) | 2.00 (7.69) | 1.00 (3.85) |  |  |
| **How likely or unlikely are you to use the PR program again?** | TelePR | 40.00 (72.73) | 12.00 (21.82) | 2.00 (3.64) |  | 1.00 (1.82) | 0.9327 |
|  | SPR | 21.00 (77.78) | 5.00 (18.52) | 1.00 (3.70) |  | 0.00 (0.00) |  |
| **How likely or unlikely are you to recommend the PR program?** | TelePR | 51.00 (91.07) | 3.00 (5.36) | 1.00 (1.79) | 1.00 (1.79) | 0.00 (0.00) | 0.6428 |
|  | SPR | 24.00 (88.89) | 2.00 (7.41) | 0.00 (0.00) | 0.00 (0.00) | 1.00 (3.70) |  |
| **How interested or uninterested would you be in continuing with PR?** | TelePR | 33.00 (73.33) | 10.00 (22.22) | 1.00 (2.22) | 1.00 (2.22) |  | 0.8966 |

**Appendix S1**.

We included several steps in our protocol to mitigate missing data. First, staff received training and routine oversight to minimize errors in data collection and storage. Second, for our primary outcome we anticipated that due to the wide geographic spread of our health system and the fact that the majority of patients stay within our health care system for their care, we would have near-complete data for this primary outcome. In addition, the health system has access to all the hospital admissions in the larger geographic area surrounding our health system in the event that patients were seen elsewhere. All patients were called on a monthly basis to ask about readmissions, which included any readmissions happening outside of the tristate area. Finally, the MZRCF method of obtaining consent for study enrollment allowed for the collection of prospective data on patients even if they choose to not adhere to either type of PR, via health record review.

*Statistical methods to handle missing data.* Despite our best efforts to prevent missing data, missing data will inevitably occur. Missing data was handled using multiple imputation. The three sets of models were constructed separately using *1)* complete data only, and *2)* missing data imputed through multiple imputation, where the missing data mechanism was assumed to be missing at random. No variable selection procedures were applied to models 1 and 2. However, variable selection procedures were applied to model 3, in order to select the significant core variables for the final model, while retaining arm, race and hospital. This process was performed differently when only complete data was used (i.e., case-wise deletion of missing data), as compared to when missing data were imputed through multiple imputation (i.e., no exclusion of cases). When variables for which complete data was available were used in model 3, the variables to be included in the final model were those that had standardized differences between the two arms greater than 0.1, and p-value <0.05 in the univariate analyses. Variables were then selected into the final model using backward selection, while forcing arm, race, and hospital to remain in the model.

When multiple imputation was used, we imputed five complete data files. In each of those data files, the standardized differences for each variable were computed. In order to select variables for inclusion in the univariable analyses, we selected variables corresponding to the median of the five standardized differences. A given variable was only included if its median standardized difference (across all five imputations) was greater than 0.1. After all the candidate variables were identified in the univariate analyses, backward selection was performed by removing the least significant variable one at a time until the pooled p-values for all the variables were less than 0.05, while forcing arm, race and hospital in the model.

In addition to the three main models, we also explored the potential interaction between treatment arm and each of race and clinical site (applies to models 2 and 3 only).

**Table S2.** Modified Borg Scale administered to participants in both TelePR and SPR at Day 1 (session 1) and 8-weeks (session 16).

|  | **Day 1** | | | **8 Weeks** | | | **Change 8 weeks - Day 1** |
| --- | --- | --- | --- | --- | --- | --- | --- |
|  | **Pre-Session** | **Post-Session** | **Pre-Post Session Change** (n=62 TelePR; n=37 SPR) | **Pre-Session** | **Post-Session** | **Pre-Post Session Change** (n=59 TelePR; n=34 SPR) | **Difference in Pre-Post Session Change 8 weeks vs. Day 1** |
| **TelePR** (n=62), Mean (±SD) | 2.82 (2.61) median:3.00 | 6.61 (2.71) median:7.00 | 3.79 (2.67) median:3.00 | 3.12 (2.88) median:7.00 | 6.91 (2.90) median:7.00 | 3.79 (2.78) median:3.00 | 0.14 (3.55) median:0.00  p^†^= 0.7700 |
| **SPR** (n=37), Mean (±SD) | 2.24 (2.91) median:1.00 | 4.53 (2.82) median:5.00 | 2.29 (2.37) median:2.00 | 2.04 (2.09)  median:2.00 | 4.27 (2.09) median:4.00 | 2.23 (1.48) median:2.00 | 0.31 (2.35)  median:0.00  p^†^= 0.5100 |
| **p-value**^*^ | Between arm  p^*^=0.2100 | Between arm | Between arm  p^*^=0.0100 | Between arm  p^*^=0.1100 | Between arm | Between arm  p^*^=0.0200 | Between arm difference in change in 8 weeks vs. Day 1  P^*^= 0.5600 |
| Note: Higher score indicates more dyspnea  *Nonparametric Wilcoxon rank sum test/two-sample t-test  ^†^Nonparametric Wilcoxon signed rank test/Paired t-test | | | | | | | |

**Figure S4**. Resistance Level on Bicycle (TelePR) and treadmill (SPR) from Day 1 (start of PR) to Day 16 (completion of PR).


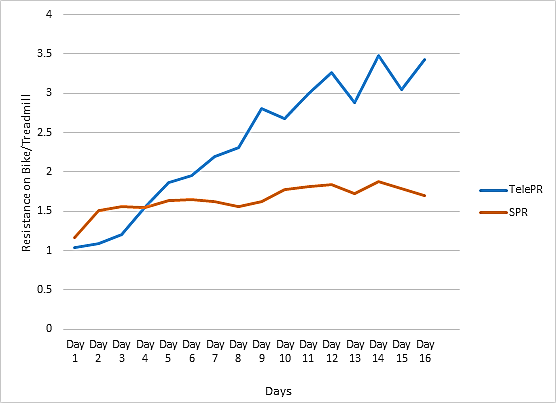


The p-value for change in resistance level of exercise at Day 16 (i.e., at 8 weeks) compared to Day 1 was 0.0001 within both the TelePR and SPR arms.

The p-value comparing increase in resistance level over time for TelePR versus SPR was 0.0033.

**Figure S5.** Duration on Bicycle (TelePR) and treadmill (SPR) from Day 1 (start of PR) to Day 16 (completion of PR).


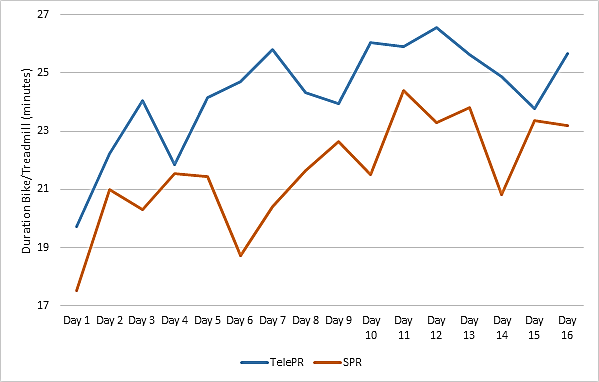


The p-value for TelePR change in duration of exercise on Day 16 compared to Day 1 was 0.028.

The p-value for SPR change in duration of exercise on Day 16 from Day 1 was 0.0313.

The p-value comparing both TelePR and SPR was 0.684.

**Table S3. Longitudinal Outcomes.** Surveys administered over the entire follow up period directly before and after the PR program (for those who participated in at least one PR session or agreed to be followed over time) (i.e., Day 1, and at 8 weeks); and then at 6 months, and 12 months.

|  |  | **Day 1** | **8 Weeks** | **6 Months** | **12 Months** |
| --- | --- | --- | --- | --- | --- |
| **COPD Assessment Test (CAT)**  Maximum Score: 40 (lower score denotes improvement) | TelePR (n=62), Mean (±SD) | 22.37 (8.35) median: 24.00 | 19.91 (7.94) median: 21.00 | 21.12 (9.55)  median:23.00 | 23.00 (8.26)  Median: 23.00 |
|  | SPR (n=37), Mean (±SD) | 20.89 (8.53) median: 24.00 | 20.18 (8.41) median: 20.00 | 23.07 (7.54)  median:22.5 | 20.09 (7.70)  Median: 21.50 |
|  | **p-value^*^** | 0.4031 | 0.9473 | 0.3151 | 0.1485 |
|  | **Overall p-value**^†^ | ARM: 0.7735, Time <.0001, Time* ARM: 0.3408 | | | |
|  |  | | | | |
| **Modified Medical Research Council Scale (MMRC)**  Maximum Score: 4 (lower score denotes improvement) | TelePR (n=62), Mean (±SD) | 2.46 (1.16) median: 3.00 | 2.28 (1.14) median: 2.00 | 2.15 (1.30)  Median: 2.00 | 2.42 (1.20)  Median: 3.00 |
|  | SPR (n=37), Mean (±SD) | 2.24 (1.04) median: 2.00 | 2.24 (1.28) median: 2.00 | 2.48 (1.18)  Median: 2.00 | 2.45 (0.91)  Median: 3.00 |
|  | **p-value^*^** | 0.227 | 0.8551 | 0.2886 | 0.9746 |
|  | **Overall p-value**^†^ | ARM: 0.4065, Time <.0001, Time* ARM: 0.3714 | | | |
|  |  | | | | |
| **PROMIS: Depression**  Maximum Score: 20 (lower score denotes improvement) | TelePR (n=62),  Mean (±SD) | 8.35 (4.21) median: 8.00 | 7.97 (4.48) median: 6.50 | 8.60 (4.28)  Median: 8.50 | 8.29 (4.32)  Median: 6.00 |
|  | SPR (n=37),  Mean (±SD) | 7.38 (4.24) median: 6.00 | 7.85 (3.78) median: 7.00 | 9.25 (4.20)  Median: 9.00 | 8.47 (4.25)  Median: 7.00 |
|  | **p-value^*^** | 0.2533 | 0.836 | 0.3813 | 0.6905 |
|  | **Overall p-value**^†^ | ARM: 0.8225, Time 0.1050, Time* ARM: 0.4088 | | | |
|  |  | | | | |
| **PROMIS: Fatigue**  Maximum Score: 20 (lower score denotes improvement) | TelePR (n=62),  Mean (±SD) | 11.77 (4.95) median: 10.50 | 10.43 (4.53) median: 10.50 | 11.38 (4.90)  median: 11.00 | 10.92 (4.54)  median: 10.00 |
|  | SPR (n=37),  Mean (±SD) | 9.70 (4.47) median: 9.00 | 10.88 (4.21) median: 11.00 | 11.43 (3.99)  median: 12.50 | 11.50 (4.68)  median: 11.50 |
|  | **p-value^*^** | 0.0707 | 0.5933 | 0.8555 | 0.6759 |
|  | **Overall p-value**^†^ | ARM: 0.8882, Time 0.5030, Time* ARM: 0.0152 | | | |
|  |  | | | | |
| **PROMIS: Informational Support**  Maximum Score: 20 (higher score denotes improvement) | TelePR (n=62),  Mean (±SD) | 14.95 (5.24) median: 17.50 | 16.10 (4.61) median: 17.00 | 15.31 (4.72)  median: 16.00 | 15.39 (4.57)  median: 16.00 |
|  | SPR (n=37),  Mean (±SD) | 15.76 (4.79) median: 16.00 | 15.64 (4.70) median: 17.00 | 16.07 (4.12)  median: 16.50 | 16.28 (4.40)  median: 17.50 |
|  | **p-value^*^** | 0.4224 | 0.5797 | 0.5516 | 0.3379 |
|  | **Overall p-value**^†^ | ARM: 0.3906, Time: 0.5838, Time* ARM: 0.5378 | | | |
|  |  | | | | |
| **PROMIS: Social Isolation**  Maximum Score: 20 (lower score denotes improvement) | TelePR (n=62),  Mean (±SD) | 7.97 (4.34) median: 6.00 | 8.66 (4.56) median: 7.00 | 7.42 (4.28)  median: 6.00 | 7.53 (3.89)  median: 6.00 |
|  | SPR (n=37),  Mean (±SD) | 7.39 (3.77) median: 7.00 | 8.79 (4.54) median: 8.00 | 8.54 (4.61)  median: 8.00 | 7.97 (4.37)  median: 7.00 |
|  | **p-value^*^** | 0.7009 | 0.7768 | 0.2565 | 0.6922 |
|  | **Overall p-value**^†^ | ARM: 0.9995, Time 0.0552, Time* ARM: 0.4242 | | | |
|  |  | | | | |
| **PROMIS: Instrumental Support**  Maximum Score: 20 (higher score denotes improvement) | TelePR (n=62),  Mean (±SD) | 15.60 (4.90) median: 17.50 | 15.53 (5.01) median: 17.00 | 15.88 (4.86)  median: 18.00 | 16.16 (4.58)  median: 18.00 |
|  | SPR (n=37),  Mean (±SD) | 15.70 (5.24) median: 17.00 | 16.00 (4.37) median: 18.00 | 15.46 (5.10)  median: 17.50 | 15.44 (5.36)  median: 17.50 |
|  | **p-value^*^** | 0.8612 | 0.9628 | 0.7948 | 0.5590 |
|  | **Overall p-value**^†^ | ARM: 0.8951, Time 0.8563, Time* ARM: 0.4818 | | | |
|  |  | | | | |
| **PROMIS: Anxiety**  Maximum Score: 20 (lower score denotes improvement) | TelePR (n=62),  Mean (±SD) | 8.71 (4.21) median: 8.00 | 8.29 (4.40) median: 7.00 | 8.58 (4.43)  median: 8.00 | 8.75 (4.23)  median: 8.00 |
|  | SPR (n=37),  Mean (±SD) | 8.38 (4.70) median: 8.00 | 8.85 (4.14) median: 8.00 | 9.14 (4.12)  median: 9.00 | 9.00 (4.52)  median: 8.00 |
|  | **p-value^*^** | 0.6369 | 0.3217 | 0.4075 | 0.8540 |
|  | **Overall p-value**^†^ | ARM: 0.8121, Time 0.9318, Time* ARM: 0.7386 | | | |
|  |  | | | | |
| **PROMIS: Companionship**  Maximum Score: 20 (higher score denotes improvement) | TelePR (n=62),  Mean (±SD) | 14.26 (4.76) median: 14.00 | 14.88 (4.68) median: 16.00 | 13.87 (4.78)  median: 15.00 | 14.39 (4.60)  median: 14.00 |
|  | SPR (n=37),  Mean (±SD) | 14.14 (4.93) median: 14.00 | 13.27 (4.42) median: 14.00 | 13.82 (4.16)  median: 13.50 | 14.53 (4.93)  median: 16.00 |
|  | **p-value^*^** | 0.9275 | 0.073 | 0.8196 | 0.8725 |
|  | **Overall p-value**^†^ | ARM: 0.7344, Time 0.7492, Time* ARM: 0.3613 | | | |
|  |  | | | | |
| **Continuation of Exercise**  (Has participant continued to exercise: “Do you continue to exercise after completing PR? yes/no”) | TelePR (n=62),  N (%) |  |  | 5 (13.16%) | 9 (22.50%) |
|  | SPR (n=37),  N (%) |  |  | 4 (19.05%) | 3 (12.50%) |
|  | **p-value^*^** |  |  | 0.5469 | 0.3211 |
|  | **Overall p-value**^†^ |  |  | ARM: 0.8686, Time 0.8909, Time* ARM: 0.2823 | |
| *Two-sample t-test or Wilcoxon rank sum test  ^†^p-values for each variable from repeated measures analysis of variance “MMRMA” (p-value for Arm: if there was difference between two arms in the outcome variable, p-value for Time: if the outcome changed from 8 weeks to day 1; p-value for Arm*Time: if the changes of the outcome variable over time differ between the two arms | | | | | |

**Appendix S2.** Overview of the Interviews and focus groups pre- and post- RCT

**Methods:**

Post-RCT qualitative data analysis

After discovering the large number of participants who did not adhere to the study protocol, we conducted a qualitative study to determine factors impacting both high and low adherence among a subsample of individuals enrolled in the study. The sub-study involved both structured interviews and focus groups. Questions were developed to provoke open ended discussion focused on specific domains of interest to the study team: a) participants’ initial reactions to being approached by a study recruiter, b) specific experiences and feelings about participating in either TelePR or SPR, c) barriers to participating in either TelePR or SPR, and d) post-intervention impact on participant’s ongoing health maintenance. Interview questions were tailored to the intention to treat categories and randomization groups (TelePR or SPR). Interviews were conducted in either English or Spanish depending on the participant’s preference. Participants who completed TelePR and SPR as well as participated in the interviews, also then participated in the focus groups, except for 1 participant who was not interviewed due to scheduling conflicts.

*Sampling strategy*

For the Interviews, participants for the sub-study were recruited from among the 209 participants who had been enrolled into the wider study, and represent those randomized to either TelePR or SPR, further stratified from among the above stated intention to treat categories for both. Sample sizes for the qualitative study were based on prior literature discussing data saturation for thematic analysis which states that data saturation for interviews can be achieved with a sample size of approximately 12 individuals per homogenous group, and that roughly 80% of all themes could be discovered in 2-3 focus groups. For the one- on-one interviews, we were interested in adherence behaviors. This meant approximately 12 people for each intention to treat group (approximately 36 people), roughly split between AA and Hispanic participants. For the focus groups, we were interested in factors enabling successful completion of the program (i.e., continued within the PR program for 8-weeks regardless of whether they attend every session). Therefore, we recruited only individuals who had completed the program – this meant 2 focus groups in total (1 for TelePR and 1 for SPR) with 5-8 people roughly split between AA and Hispanic participants.

*Data collection methods (provide rationale for refinements in the context of research questions)*

All participants recruited into the sub study were re-consented to participate in these interviews as they were not part of the initial RCT consent. All interviews and focus groups were audio-recorded, transcribed by a professional medical transcribing company including translating from Spanish to English when necessary. The focus groups were conducted in English, all participants, including the Hispanic participants were proficient in both Spanish and English. All audio recordings and transcripts were stored in PHI HIPAA compliant folders.

*Approach, research paradigm, and guiding theory for Interview Data Analysis*

Our qualitative analysis employed a hybrid design drawing on several well-established approaches and theories, specifically: 1) deductive analysis or a hypothesis-based approach. This was driven by a) the overall goals of our sub-study to better understand adherence to the study protocol, and was used to guide development of the interview and focus groups guides, and b) theoretical frameworks rooted in the Common Sense Model which asserts that individuals use both cognitive and affective processes to identify their illness, and understand the causes, treatment options, and timelines of their illness trajectories; and the Alternative Knowledge Framework stating that individuals often understand their illnesses and make decisions regarding their care based on non-biomedically-based information including culture and prior lived experiences; 2) inductive analysis allowing themes and ideas to generate through iterative readings of the transcript texts. We also used 3) narrative synthesis to diagram the various participation scenarios (i.e., possible courses of action that participants might take once being recruited into the study). For this, a Journey Diagram was created, which subsequently served to organize the primary themes used in the codebook and in the coding database (Nvivo). 4) We also used the constant comparison method, commonly used in deductive approaches such grounded theory [38-40], that allows for the iterative refinement of themes into a final set of codes that can then be used to code all of the transcripts. Finally, 5) we engaged in a process of reflexivity, whereby discussions about how the backgrounds and points of view of the qualitative research team could impact the interpretation of the data. These discussions were part of the weekly qualitative research team meetings.

*Interview Data Qualitative Analysis*

Thematic analysis was undertaken by 3 members of the study team comprised of a first-generation Latina research coordinator, a PhD level Afro-Caribbean Industrial/Organizational psychologist/health disparities researcher, and a PhD level Caucasian, medical anthropologist/qualitative analyst whose research focused on chronic lung disease patients. This team met weekly for 2 months to review the transcripts and discuss the themes they discovered, incorporating reflexive discussions about the impact of their respective backgrounds on their interpretation of the data. Each week this team would read 5 transcripts and prepare a list of themes to discuss until saturation was reached. After discussing 15 transcripts, saturation of themes was reached. The team agreed to read an additional 5 transcripts to ensure saturation. At that point, each team member created a final list of themes. The team then went through a process of iteratively refining and discussing the themes using the constant comparison method until themes were narrowed down to one final list of themes with agreed upon nomenclature. The themes were further organized according to the possible outcome’s scenarios presented in the Journey Diagram. This allowed us to distinguish between primary themes that may have been only relevant to either TelePR or SPR participation. Additionally, the decision was made to include an additional primary theme to cover statements that were applicable to either SPR or TelePR. (e.g., one barrier to participation was feeling too sick to exercise; this is neither unique to TelePR nor SPR). From this final set of themes, a codebook was developed. This codebook consisted of the list of themes and for each theme, a definition, exemplary quote, and exclusion and inclusion criteria was provided. Using this codebook, the transcripts were coded in Nvivo Pro 12 by 2 members of the qualitative research team. Both coders kept lists of items that required clarification to be discussed at weekly coding meetings. Coding comparisons were run in Nvivo initially after both coders finished coding the same 5 transcripts each week and then gradually moving to 10 transcripts per week. A Kappa coefficient of less than 0.85 during the weekly check in meetings required discussion among the coders to resolve discrepancies and reach agreement.

**Results**

Focus Group Results from CAB meetings before the start of the RCT

Based on the feedback of CAB members, and of patient engagement, several changes were made to the recruitment strategy and strategies to increase retention. No changes were made to the content of the PR sessions, including exercise type and duration, as well as what education was provided. Examples of results of CAB feedback and patient engagement include: (1) *Measures to improve recruitment*: development of brochures to give out to patients at the time of recruitment; creating patient testimonial videos to show during recruitment; culturally tailoring the videos with different music and Hispanic/African American actors; connecting with local pulmonologists to help recruit patients during outpatient visits; connecting with the hospitals’ respiratory therapist to inform the patients of the study prior to our recruiters approaching during their hospital admissions; using the term of movement therapy instead of pulmonary rehabilitation exercise, and making sure that potential participants knew the exercises would be tailored to their particular level of capacity. The researcher team included weekend and even hours to make sure family members could be easily asked during recruitment (2) *Measures to enhance retention:* connections with community centers to provide TelePR for those patients that did not have space for equipment; distributing a monthly newsletter for enrolled participants which provided information about relevant events happening around NYC, and; addition of a mircokey to the telehealth tablet computer to make it easier for older patients to turn on/off and to access the features needed; a laminated “how to” sheet attached to the equipment. Detailed qualitative results are presented below.

The first two CAB meetings were convened as focus groups. Discussions centered on the adaptation of the TelePR program, from both consumer and provider perspectives. Consumer perspectives mainly focused on cultural tailoring, whereas provider perspectives focused on equipment functionality (e.g. can a frail, older patient sit on the bike?). The first meeting began with a general discussion of the needs of COPD underserved patients; multiple challenges were identified, including factors that affect access to PR. These factors included insurance payment for PR, repeated hospitalization, medication management, and comorbidities. After the general need’s assessment discussion, the principal investigators presented a demonstration of the initial version of the rehabilitation equipment including the tablet to be used, vital sign equipment and the bicycle. A total of 5 major themes emerged from qualitative analyses of community discussions: equipment changes, recruitment processes, study logistics, self-efficacy, and access. The identification of themes was instrumental in understanding the concerns of patients and other stakeholders in adapting the PR home intervention for acceptability for patients with COPD from underserved communities. The quotes below were from several CAB meetings and due to several people speaking in the focus group recording the quotes cannot be attributed to a specific participant.

**Theme 1: Equipment Changes**

***Subtheme 1: Safety and Comfort***

One theme that emerged quickly was bike ergonomics: the need for a bike that was safe and comfortable for patients with relatively limited mobility. Specifically, 1 participant noted that the bike would need to be stable with a seat that patients can get in and out of easily. One patient noted:

*“You don’t have to struggle... with all the equipment, everything is set up just right for the patient to access everything.”*

Specifically, the upright nature of the bike was perceived as dangerous, as patients could more easily fall because of the unstable nature of the design; a recumbent bicycle was strongly encouraged by the CAB. A patient noted:

*“The bike is not difficult, but the bike was breaking down. The arm wasn’t good...”*

Another provider participant noted that the tablet attached to the bike was too high and needed to be at eye level. The arm, which holds the tablet, should have the ability to swivel to facilitate getting in and out of the bike and to accommodate for varying patient heights. A stable arm or surface was deemed necessary to help with transfer; choosing the voice that speaks to the patient, in the accent that is most familiar; considering the gender of the patient and their wardrobe (wearing dresses); and a wider seat with more cushioning than a typical bike to accommodate an older, larger body.

***Subtheme 2: Ability to See Vital Signs***

One patient participant discussed that he would want the ability to see his vital signs while exercising on the bike. Having access to feedback—the ability to look at vital signs—is a good teaching opportunity for participants. If patients make adjustments during exercise and see an improvement in their vitals, this feedback reinforces what they are being taught by the respiratory therapist.

**Theme 2: Recruitment Changes**

***Subtheme 1: Recruitment Brochure***

CAB patient participants recommended multiple updates to a recruitment brochure that was developed by staff. These changes included increasing the font size, testing the grade level it is written in, and checking that information is accurately translated for multiple dialects. For example, several patient members of the CAB suggested not just using the term COPD but also using the term Enfermedad pulmonar obstructiva crónica (EPOC).

***Subtheme 2: Culturally Tailoring the Recruitment Process***

A second theme that emerged from the discussion was the need to culturally tailor the enrollment process for a population that does not often receive PR or frequently participates in research.

At the third CAB meeting, the staff presented a recruitment video and a recruitment brochure in response to previous CAB recommendations to develop a recruitment video. The video used an untrained actor posing as a patient. One patient suggested including multiple patients exercising together in the video. This would show communication and support among patients. Another patient suggested having real patients in the recruitment video (as opposed to untrained actors) to make the experience look more realistic:

*“...the video was very good, but I think it will be much better when they get more people involved...they’re communicating together at the same time, it’s like...supporting each other, you know...”*

*“It didn’t impress me much. I liked the part that you played, I thought you did a very good job there. But he doesn’t really look the part... I think you want to think about incorporating the caregiver, because...it’s not just the patient... it’s the spouse (Mrs. Lopez) (*names changed to protect patient privacy) who’s been watching... been part of the story of Mr. Lopez, just as much as he’s been a part. Different role, but together. [Mrs. Lopez] was very clear and very eloquent talking about this. And...[Mrs. Lopez] said that now you can’t get him off the bike... It’s an inspiration to see Mr. Lopez, but also to hear Mrs. Lopez...So I would just suggest you think about the caregiver.”*

At the second CAB meeting study enrollment was further discussed; both patients and providers suggested using a recruitment brochure and a video for enrollment that shows a patient with COPD that “looks like me” (Hispanic and African-American patients) successfully participating in PR.

**Theme 3: Study Logistics**

***Subtheme 1: Lateness Protocol***

The respiratory therapist asked the CAB what to do when a patient arrives late to the multiuser session. Both patients and providers suggested having a protocol in place for when this happens, including putting a time limit on how late they can arrive, stating that they will not receive extra time if they are late, and providing phone call reminders before the start of class:

*“When you’re on a multi-person call... if people start showing up late, everything gets disrupted, so now you’ve got a dosage issue because your one-hour session just became 45 minutes because Person 1 was there on time, Person 2 was ten minutes late...and you’ve got to redo your greetings, and you—it disrupts everything.”*

***Subtheme 2: Gradual Exercise***

The CAB discussed the patient educational process in using the bike for exercise. Specifically, providers recommended that the exercise be presented as graded tasks (i.e., starting simple with 1 or 2 instructions and adding on over time). Given that the COPD bike is different from an ordinary bike, requiring an extended, longer, and more gradual learning period, it is essential for patients to be comfortable with what they are doing and understand what is going on:

*“Exactly, you cannot just throw it at one time, you gotta educate them, like from A to B…you gotta teach them step by step.”*

**Theme 4: Self-Efficacy**

***Subtheme 1: Communication***

CAB members discussed the importance of being able to communicate effectively during exercise. This communication between both the respiratory therapist and patient is to foster learning and understanding of the program and how patients are reacting to the movements. Our patient partner shared:

*“Yes, absolutely. I am able to hear him [the RT] loud and clearly, we’re able to interact and switch between bike and exercises and warm-up, and I ask him to (put on his?) blood pressure cuff and his pulse oximeter, everything is—we are able to do pretty much everything.”*

***Subtheme 2: Motivation***

One participant discussed how motivating it is for them to see others succeeding on the bike, particularly patients who are similar in terms of age, condition, ability, etc. He noted that seeing others succeed gives him the confidence that he too can successfully participate in the program. Another patient commented on how they were personally motivated to do more things because of the program:

*“The way [Mr. Lopez] here was doing the bike, when we see the video, we get motivated seeing him doing it...the way he’s breathing is excellent according to what I saw there.”*

*“It got me out of my room with my anxiety and my depression—it motivated me to do other things that I didn’t do before.”*

***Subtheme 3: Health Control***

There was additional CAB discussion about regaining control of one’s health resulting from perceived improvements in health status as a result of exercise. The discussion reflected that patients have a better understanding of what to do to improve their health. Patients who completed the program commented that they were able to see improvements from the exercise and use what they have learned outside of the program:

*“I liked them a lot, I wanted to see if this worked for me. It wasn’t easy at first because I wasn’t used to it, but now I can even do them on my own.”*

*“It was hard in the beginning. Because when you don’t exercise for a long time and then you start—it is stressful but then gets easier. I was always allowed to rest during the sessions. I felt an 80% improvement. My chest isn’t that tight and I can breathe through my nose when I do the breathing exercises that I learned in the program.”*

***Subtheme 4: Importance of Coaching and Presence in the Home***

A CAB member (patient advocate) remarked that, given the importance of culturally congruent social interaction within the Hispanic community, having a combination of in-person interaction in addition to Web-based interaction (via technology)—rather than exclusively online—is more meaningful and preferable to patients.

**Theme 5: Access**

On multiple occasions, the CAB discussed the benefit of having access to PR right in one’s home. Given that for this patient population, it is not always feasible to travel to therapy, having this access allows patients to continually participate in exercise—an important part of maintaining quality of life. One patient also noted that they felt more comfortable being at home with this program than going out to a gym:

*“The other thing I think it’s great, because in the home...winter time, it’s snowing, raining, sleet, and all that stuff... sometimes it’s not feasible to get there. So, if you got [the respiratory therapist] on the screen, you can still do what you got to do.”*

**Interview and Focus Group Results Post-RCT**

Among participants randomized to either TelePR or SPR, we saw poor uptake of the PR program. To better understand why this might be happening, we undertook a sub-study using qualitative methods to determine factors impacting adherence to the PR program.

**Table S4**. Focus group and interviewed participant’s demographics

|  | | |
| --- | --- | --- |
| **Participant Characteristics** | **Interview N=39** | **Focus Group N=2** |
| **Age – categorical, N (%)** | | |
| 18-64 years | 20.00 (51.00) | 4.00 (40.00) |
| ≥ 65 years | 19.00 (49.00) | 6.00 (60.00) |
| Unknown or not reported | 0.00 (0.00) | 0.00 (0.00) |
| **Age – continuous, Mean (±SD)** | | |
|  | 64.10 (10.31) | 63.25 (10.33) |
| **Sex, N (%)** | | |
| Female | 27.00 (69.00) | 8.00 (80.00) |
| Male | 12.00 (31.00) | 2.00 (20.00) |
| **Ethnicity, N (%)** | | |
| Hispanic | 11.00 (28.00) | 1.00 (10.00) |
| African-American | 28.00 (72.00) | 9.00 (90.00) |
| **Arm, N (%)** | | |
| TelePR | 19.00 (49.00) | 5.00 (50.00) |
| SPR | 20.00 (51.00) | 5.00 (50.00) |
| **Study Status, N (%)** | | |
| Completed 8 weeks of PR | 20.00 (51.00) | 10.00 (100.00) |
| Followed over 8 weeks (no PR only questionnaires) | 6.00 (15.00) | 0.00 (0.00) |
| Withdrew After Clearances | 5.00 (13.00) | 0.00 (0.00) |
| Withdrew Before Clearances | 2.00 (5.00) | 0.00 (0.00) |
| Excluded After Clearances (past 30 days to start PR) | 4.00 (10.00) | 0.00 (0.00) |
| Excluded Before Clearances (past 30 days to receive clearances) | 2.00 (5.00) | 0.00 (0.00) |

Thematic analysis of the transcripts from the audio recordings showed 5 primary themes related to barriers and facilitators to participating in the study or in physical activity: *1)* Approach, Recruitment and Enrollment, *2)* Experiences Unique to Those Participants Randomized to SPR, *3)* Experiences Unique to Those Participants Randomized to TelePR, *4)* Experiences that are Relevant to either TelePR or SPR, and 5) Post Intervention PR Maintenance. At the time individuals were approached to enroll in the study, their prior level of familiarity with PR and their beliefs about PR efficacy were the biggest factors determining individual-level interest in participating in the study.  For those randomized into SPR, the security of being with other people were cited as the biggest motivating factors for participating in the program. Lack of time and transportation issues were cited as barriers to participation. For participants randomized to TelePR, factors facilitating participation included the convenience of having the exercise equipment in their homes. Barriers included inability to use the equipment and the inconvenience of having to store the equipment at home. Both SPR and TelePR participants cited the socialization, personalized exercise, and effectiveness of PR on their health as factors promoting their participation in the program. Both groups included feeling too sick to exercise and having to prioritize other co-morbidities over PR (e.g. cancer treatment, dialysis) as primary barriers to participation. Finally, after completing either PR or TelePR, several participants stated that they were continuing to engage in some form of exercise (e.g. walking). Those who were no longer engaging in physical activity cited a lack of access either to a PR facility or to the exercise equipment as barriers to continued physical activity.

1) Approach, Recruitment and Enrollment: Among the 39 participants that we interviewed, 3 people stated that they had not known they had COPD at the time of their hospitalization, 7 participants expressed a lack of familiarity with how to treat COPD in general, and 11 stated that they were unfamiliar with PR. For some, the fact that their clinician never recommended PR was a factor in their decisions not to participate. As stated by one participant:

“*Like I said, my doctor never told me anything about that [PR]. I mean, he could tell his patients when they have severe -- chronic breathing, respiratory infections, and things like that. He should advise his patients to just give it a try. If he would have told me to go, I would have went”* (Hispanic female with COPD, age 62 years).

For those who ultimately did participate in the program, factors facilitating their decisions to participate included the belief that PR was effective and having the support of their physician. For example:

“*My doctor walked in and said, she's a good candidate for the program. Put her in it”* (African-American female with COPD, age 67 years).

Ultimately, initial decision making about whether to participate in the program was impacted by the extent to which individuals approached for the study perceived that PR would be helpful to them for treating their COPD.

2) Experiences Unique to those Participants Randomized to SPR: For the 20 randomly selected sub-study participants randomized to SPR, those who ultimately completed the 8 week program frequently cited as pros: 1) peer motivation, 2) PR-related education, and 3) the security of having the respiratory therapist present. For those who did not complete the SPR program, barriers to participation were: 1) lack of transportation, 2) financial difficulties (e.g., inability to cover co-payments), and 3) scheduling conflicts.

3) Experiences Unique to those Participants Randomized to TelePR: Among those in the TelePR Arm, facilitators to participation included the convenience of being able to participate from home, particularly for those participants unable to leave the house at all due to their COPD and/or co-morbidities. For example:

“*I have a very difficult time walking.  I get out of breath very rapidly. So this was a godsend, or to me an excellent idea, in terms of doing it in-house. If I had to roll into a central location and do it, more than likely I would have not been in the program or else dropped out*” (African-American male with COPD, age 76 years).

Additional facilitators to TelePR included: learning from the RT how to perform physical and breathing exercises and medication management (e.g.: “*He [the RT] also would show me via the tablet, how to use the medication.  You know, often you don't get to know how to do something very well, because the doctors often don't have the time to show you what to do with the medication they give you*” (Hispanic female with COPD, age 66 years)). Participants also formed close relationships with their RTs.

For those who did not complete the TelePR program, barriers included: 1) difficulty using the exercise equipment, 2) housing issues - both not having a permanent residence, or not having a large enough space for equipment, and 3) scheduling conflicts.

4) Experiences Relevant to Either TelePR or SPR: There were several facilitators and barriers to participation that were relevant to both SPR and TelePR.

among both arms, *frequently cited as pros included*: 1) Tailored exercise; 2) the socialization and peer support during the sessions, 3) effectiveness of the PR program; those who completed the program frequently cited the benefits in their health; and 4) increased self-efficacy with many participants stating that PR helped them to feel more in control of their health.

For **those who did not complete either the TelePR or SPR program**, barriers cited were*:* 1) the “Catch 22” of participants recognizing the likely benefits of PR but feeling too sick to participate; 2) needing to prioritize other comorbidities over their COPD. Here individuals wanted to participate, but felt their other illnesses were a priority (e.g., diabetes, kidney disease, and breast or lung cancer). Many participants felt that the need to attend PR sessions added to their already high treatment burden. For others, PR sessions conflicted with other medical appointments (e.g. chemotherapy and hemodialysis); 3) participants believing they could manage their own health regimens including other forms of exercise, taking medications prescribed by their clinicians, or using alternative remedies; 4) socioeconomic factors: jobs, childcare, lack of insurance, lack of family support, 5) a history of poor adherence to medical treatment in general: in many cases, individuals describe prior health histories of poor adherence to medical treatments, poor health behaviors including smoking, dislike of exercise, “being lazy”, and not following up with doctor’s appointments. In this way, lack of participation in the PR program was congruent with the prior health behaviors of such individuals; 6) lack of belief in PR efficacy or believing that they no longer needed PR once they began to feel better. Others discussed alternative treatments linked to cultural norms, and prior lived experiences for their lack of participation in the program.

5) Post Intervention PR maintenance: For those in both TelePR and SPR who completed the 8-week program, we asked about current physical activity. Eighteen people interviewed in the sub-study remained engaged in PR-related exercises or breathing techniques learned from PR. There were 14 participants who stated that they had not continued physical activity, although their health had declined as a result. Participants frequently stated that they would have liked to continue attending the PR sessions (both TelePR and SPR), but that they were unable to due to transportation and financial difficulties. Six people stated feeling abandoned by the program and felt the program had been taken away from them. As stated by one participant: “They give the equipment in order to help you, and then they take it back from you. That breaks the morale of the whole program, you know. It’s like building you up and breaking you down” (African-American male with COPD, age 67 years). In this way, those participants who liked the program and believed that they had benefitted from it, were unable to continue the benefits of PR once their time in the study had ended.

**Appendix S3.**

Of the 281 patients who were open to participation in the study, 11 were excluded because of dementia and 4 were excluded because the inpatient PFT results excluded COPD (before randomization). In total, 266 participants were randomly assigned (n = 131 to the TelePR arm and n = 135 to the SPR arm). Of the 131 randomly assigned to TelePR, 20 were subsequently excluded after randomization because they no longer met inclusion criteria (10 patients were ineligible due to PFT results indicating no COPD; 6 were too unstable medically to participate in PR; 3 became unable to ambulate or exercise; and 1 later stated they did not consider themselves African American). Of the 135 patients randomly assigned to SPR, 37 were excluded after randomization because they no longer met inclusion criteria (19 patients were ineligible due to PFT results indicating no COPD; 13 became too unstable medically to participate in PR; 4 became unable to ambulate or exercise; and 1 could not follow directions required for exercise participation). Therefore, in total, 209 participants were randomly assigned and were included in the ITT analysis that examined the comparative effectiveness of referral to TelePR vs SPR. Of the 209 participants, subanalyses were conducted for 138 who obtained all clearances and therefore were able to participate in PR and for 85 who agreed to participate in PR sessions and sat on the ergonomic stationary bike at least once.
